# Safeguarding diet quality in a changing climate: a scoping review

**DOI:** 10.3389/fnut.2026.1833758

**Published:** 2026-05-28

**Authors:** Beaula Mutonhodza, Melody Ndemera, Laurencia Govender, Anouk Reuzé, Mjabuliseni S. C. Ngidi, Muthulisi Siwela, Tafadzwanashe Mabhaudhi

**Affiliations:** 1 Centre for Transformative Agricultural and Food Systems, School of Agricultural Science, University of KwaZulu-Natal, Pietermaritzburg, South Africa; 2 Department of Nutrition, Dietetics and Food Sciences, University of Zimbabwe, Harare, Zimbabwe; 3 Department of Food Processing Technology, Harare Institute of Technology, Harare, Zimbabwe; 4 Discipline of Dietetics and Human Nutrition, School of Health Sciences, University of KwaZulu-Natal, Pietermaritzburg, South Africa; 5 Centre on Climate Change and Planetary Health, London School of Hygiene and Tropical Medicine, London, United Kingdom; 6 Discipline of Agricultural Management, School of Agriculture and Science, University of KwaZulu-Natal, Pietermaritzburg, South Africa

**Keywords:** climate resilient food systems, food security, malnutrition, nutrition security, planetary health, policy pathways

## Abstract

**Background:**

Climate change is increasingly recognized as a driver of declines in crop nutrient content; however, the evidence remains fragmented. This scoping review maps and synthesizes current knowledge on climate change and the nutrient quality of vegetables, legumes, and grains.

**Methods:**

A systematic online search of peer-reviewed experimental, meta-analytical, and modeling studies published between 1950 and 2024 was conducted, using standardized extraction methods to ensure consistency and quality.

**Results:**

Experimental studies (*n* = 132) and meta-analyses (*n* = 9) showed average increases in total carbohydrates (+11%; +17%), simple sugars (+12%; +11%), antioxidants (+14%; +43%), vitamin C (+12%; +6%), and vitamin E (+5%), alongside significant declines in dietary fiber (−13%), folate (−30%) and essential minerals (−3%; −8%), respectively. Modeling studies (*n* = 2) projected declines in global nutrient availability of up to −4% for protein and −3% for zinc and iron.

**Discussion:**

Staple foods that feed billions are losing key nutrients, driving a shift toward energy-dense, nutrient-poor diets that heighten hidden hunger and chronic disease risks. Warranting the need for nutrition-sensitive climate adaptation strategies.

## 1 Introduction

Climate change and malnutrition represent two converging global crises, intricately connected through agrifood systems (1). Climate change reduces crop yields and diminishes nutrient density (2, 3), thereby undermining food and nutrition security. At present, more than 864 million people worldwide face severe food insecurity, with low-income countries most affected; 71.5% of their populations cannot afford a healthy diet (4). Malnutrition, largely driven by poor diets, is the leading global health risk (5), with four billion people suffering from micronutrient deficiencies, particularly iron (Fe), zinc (Zn), selenium (Se), calcium (Ca), and folate (vitamin B9) (6, 7). Projections suggest that climate change will intensify these burdens: by 2050, an additional 175 million people may become zinc-deficient, 122 million protein-deficient, and 1.4 billion women and children iron-deficient (8). These nutrient shifts are further associated with heightened risks of diet-related non-communicable diseases, including cardiovascular disorders and diabetes (9).

Environmental stressors such as elevated CO_2_, rising temperatures, and ozone exposure are altering the nutrient composition of staple crops (e.g., rice, wheat, maize) (10, 11), while extreme weather and water stress further destabilize farming systems (12, 13). Global food systems are at a critical juncture, facing a crisis that could trigger a systemic collapse. Agricultural productivity continues to decline under climate stress, with Africa experiencing a 34% slowdown since 1961, the largest reduction globally, an especially concerning trend given that the region’s productivity has historically lagged behind global averages (14, 15). This establishes a direct causal pathway from climate stress to food and nutrition insecurity, compounded by structural barriers including high food prices, low incomes, and unequal access to diverse diets (16).

The impacts of climate change are most pronounced in vulnerable regions such as sub-Saharan Africa, where reliance on plant-based foods is high (17, 18). At the same time, plant-based diets are increasingly promoted for their benefits to planetary health (13, 19, 20). Yet, their long-term nutritional adequacy under climate stress remains uncertain due to limited, fragmented, and geographically uneven evidence (21, 22). Compounding this challenge is the inaccessibility and decline in marketing and consumption of nutrient-dense indigenous crops in favor of commercial varieties, which predisposes sub-Saharan African populations to malnutrition (23–25). A business-as-usual trajectory will further weaken an already fragile food and nutrition security landscape (2, 17, 18). Moreover, future scenarios of sustainable dietary transitions highlight both nutritional and environmental insecurities (26). Understanding these interactions is crucial for developing strategies that ensure diets are sustainable, nutritionally adequate, and healthy.

Research linking climate change to diet quality and nutrition-related health outcomes remains limited and fragmented, particularly for vulnerable populations (27–29). Evidence on how climate change alters key nutrients is scarce (2, 30). A clear policy disconnect exists, with only 13% of national policies jointly addressing climate and nutrition globally (1). This review seeks to bridge these gaps by (i) identifying climate-sensitive nutrients most vulnerable to climate change; (ii) synthesizing experimental, meta-analytical, and modeling evidence on crop nutrient alterations; and (iii) highlighting the crops and regions at greatest risk. The findings offer critical insights to inform agricultural and public health nutrition practices, thereby advancing both planetary health and human nutrition.

## 2 Materials and methods

### 2.1 Study design

This scoping review followed the PRISMA Extension for Scoping Reviews (PRISMA-ScR, 2018) to ensure transparency, reproducibility, and comprehensive synthesis of peer-reviewed literature (31). The scoping approach incorporated numerical summation (32) to map the breadth of available evidence, aligning with the exploratory nature of this topic.

### 2.2 Search strategy

A systematic search adapted from Scheelbeek et al. (33) (Supplementary Appendix 3) was conducted to identify studies examining the effects of elevated temperature (eTemp), carbon dioxide (eCO_2_), ozone (eO_3_), salinisation, contaminated water, and water stress on the nutrient quality of vegetables, legumes, and grains. Searches were conducted across major databases (Web of Science, MEDLINE, Scopus) and supplemented by screening the reference lists and reviewing Supplementary materials from previous climate-nutrition reviews (10, 11, 33, 34). Keywords combined terms related to “climate change,” “nutrient composition,” and “crop quality.” Records were managed and de-duplicated using Mendeley Reference Manager to ensure consistent citation management. The final search was conducted on 15 February 2026.

### 2.3 Eligibility

Quantitative studies published in English were included if they reported percentage changes in the nutrient content of edible crops under specified climate exposures. Eligible study types encompassed experimental research, meta-analyses, and modeling studies, with neutral, positive, and negative climate change impacts considered. Publication dates were aligned with historical climate data tracking from 1950 to 2024 (35, 36). Studies were excluded if they lacked data on edible crops, failed to specify the crop type, nutrient direction, or percentage change, or focused solely on yield without nutritional relevance.

### 2.4 Data extraction

The systematic search initially identified 274 studies on crop nutrient content. During the title and abstract screening stage, sixty-three studies were excluded because they did not meet the predefined eligibility criteria (e.g., irrelevant exposures or absence of nutrient outcomes). This left 211 full-text articles for detailed assessment. At the full-text review stage, each article was evaluated against inclusion criteria requiring quantitative evidence on the effects of climate-related exposures on the nutrient content of vegetables, legumes, or grains. Following this assessment, sixty-eight articles were excluded due to a lack of quantitative measures, unconfirmed crop types, or inedible crops. For each eligible study, data were extracted on study characteristics (location and exposure), crop type, nutrients or nutritional factors, direction, and magnitude of change (%), and reference details (Supplementary Tables 1–5). Study selection and data extraction were conducted independently by two reviewers, with discrepancies resolved by consensus to minimize bias.

### 2.5 Outcomes

Standardized thresholds defined elevated exposures: above 350 ppm CO_2_, +4 °C temperature, +25% O_3_, +25% salinity, −50% water stress, and contaminated water (33, 37). Thresholds were applied as categorical benchmarks during the synthesis. The primary outcomes assessed were the magnitude and direction of climate-induced changes in crop nutritional quality. Average percentage changes in crop nutrients were extracted or calculated from the minimum and maximum values reported in experimental studies (Supplementary Appendix 1). Nutritional gains and losses were then quantified by food group (vegetables, legumes, and grains) in alignment with food-based dietary guidelines (38). Where multiple data points existed for a given nutrient, values were averaged before calculating the overall (39).

### 2.6 Protocol registration

The protocol was not registered to PROSPERO, as the review did not involve human participants or report on direct health outcomes (40).

## 3 Results

### 3.1 Study selection

**FIGURE 1:**
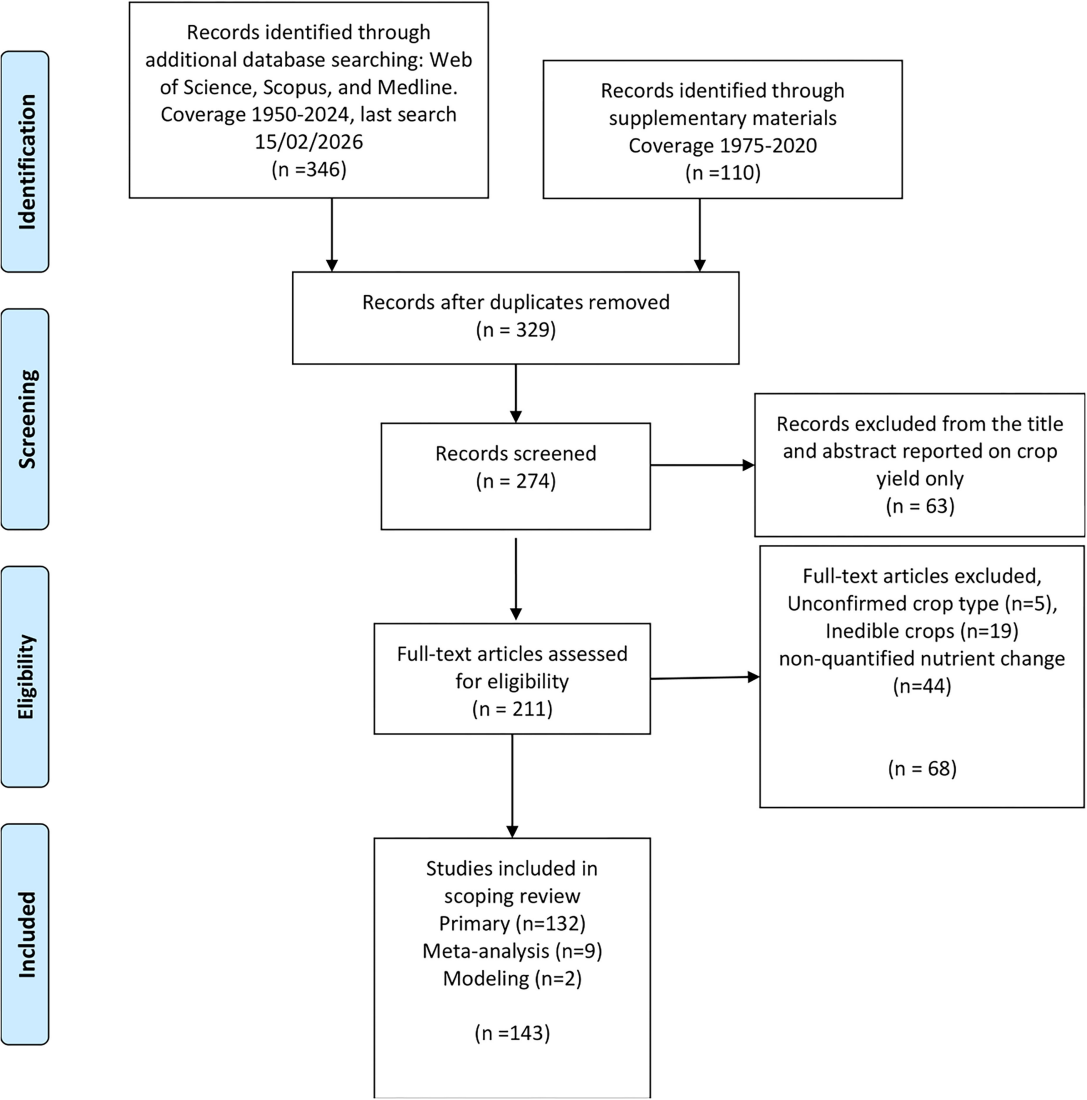
PRISMA flow diagram of study selection. From 274 records identified, 63 were excluded at title/abstract screening and 68 at full-text review, leaving 143 studies (132) experimental, 9 meta-analyses, 2 modeling) for inclusion.

In total, 143 studies were retained for the scoping review, encompassing 132 primary experimental investigations, nine meta-analyses, and two modeling studies (Figure 1).

### 3.2 Characteristics of studies included in the scoping review

The included studies spanned five continents: Africa, Asia, Europe, Oceania, and the Americas, providing broad coverage of climate zones, crop types, and farming systems. Europe had the widest country representation (*n* = 14), reflecting strong research engagement and data availability in that region. Asia and Africa followed (*n* = 7 each), indicating growing interest in climate–nutrition dynamics, while China contributed most of the individual studies (*n* = 15), highlighting a concentrated research effort on climate impacts (Supplementary Tables 1–3). Six key climate change exposures were considered: three linked to elevated climate conditions (CO_2_, temperature, O_3_) and three water-related factors (stress, salinity, contamination), with eCO_2_ being the most frequently reported. Research often combined exposures (e.g., eCO_2_ with eTemp or eO_3_). Vegetable-focused studies examined a broader range of environmental factors, whereas those on legumes and grains focused on eCO_2_ and eTemp. Fruits (*n* = 2) and C4 crops such as maize (*Zea mays*, *n* = 4) and sorghum (*Sorghum bicolor*, *n* = 2) were understudied, underscoring a research gap.

**FIGURE 2:**
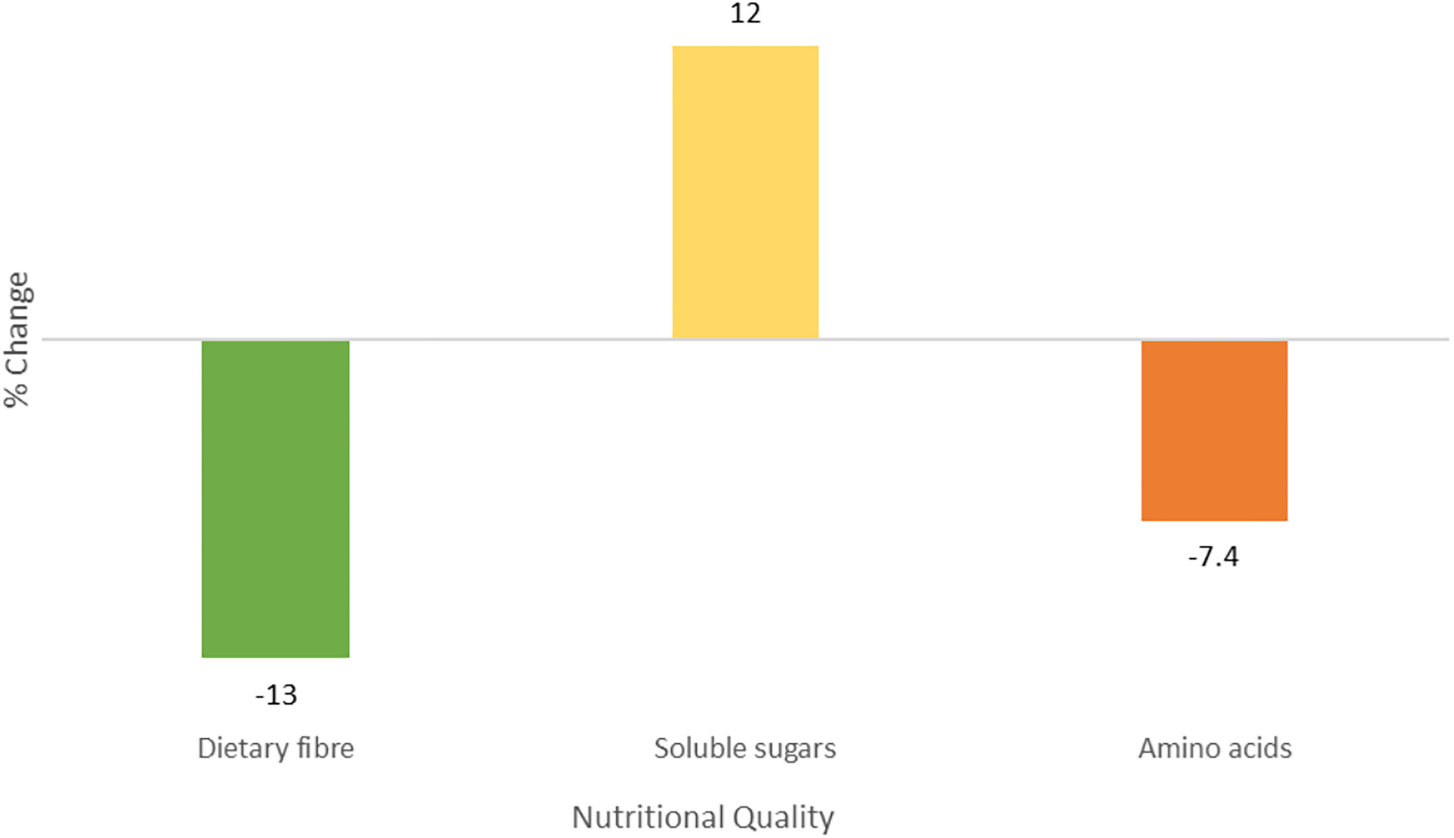
The figure is based on 82 experimental studies of vegetables, legumes, and grains, where both positive and negative climate impacts on nutritional quality parameters were observed. Percentage changes reflect crop responses to elevated exposures: CO_2_ (> 350 ppm), temperature (+4 °C), ozone (+25%), salinity (+25%), water stress (- 50%), and contaminated water. Multiple data points for each nutrient parameter were averaged. Error bars represent the standard error of the mean: phytochemicals (*n* = 59), Ca (*n* = 29), K (*n* = 22), Mg (*n* = 20), Zn (*n* = 56), Fe (*n* = 55), Mn (*n* = 13), P (*n* = 5), S (*n* = 2), vitamins (*n* = 38), protein (*n* = 10), and carbohydrate (*n* = 3). Error margins reflect discordance between field and greenhouse experiments, which were considered jointly.

Nutrient analyses across the reviewed studies encompassed macronutrients, micronutrients, phytochemicals, and antinutritional factors. Iron and Zn were the most frequently assessed minerals (*n* = 55 and 56, respectively). Phytochemicals were predominantly reported in vegetable (*n* = 59) focused research (Figure 2). Studies on vitamins, particularly B-complex (*n* = 1), and amino acids (*n* = 1), were limited (Supplementary Tables 1–5), emphasizing the need for longitudinal research to track nutrient transitions over time.

### 3.3 Differential crop responses to climate-related environmental stressors

Mixed effects of environmental stressors were observed across crops: eighty-two studies reported both positive and negative climate change impacts, while fifty reported neutral effects. Elevated CO_2_ and warming generally led to declines in essential nutrients such as Fe and Zn, but remarkably increased heavy metals (Manganese, Molybdenum, Chromium, Nickel, Cadmium, and Lead) concentrations by 9.2 to 72.9% (41, 42) in rice (*Oryza sativa*) and wheat (*Triticum aestivum*) (41). This exacerbates the risk of heavy metal contamination in grain. In contrast, vegetables and legumes exhibited positive nutritional shifts, with increases in Fe, vitamin C, flavonoids, and antioxidants (33, 43). However, the combined effects of eTemp and eCO_2_ negated these benefits in major staples such as wheat and soybeans (*Glycine max*) (Supplementary Table 5), demonstrating the complexity of compound stressors.

Additional nutrient modulation was also noted: eO_3_ boosted vitamin C in leafy greens, water stress enhanced carotenoids and flavonoids in fruit vegetables, and salinity increased carotenoid levels in tomatoes (*Solanum lycopersicum*) (33). Gains in micronutrient composition were noted in vegetables irrigated with recycled water, substantially in cabbage (*Brassica oleracea*: Fe +67%), onion (*Allium cepa*: Fe +54%, Zn +31%), spinach (*Spinacia oleracea*), and lettuce (*Lactuca sativa*) (43).

### 3.4 Crop-specific sensitivities and adaptive potential under climate change

Meta-analysis and modeling studies reveal significant reductions in the protein content of grains and legumes under eCO_2_ conditions (44, 45). Among grains, rice and wheat are the most affected, with protein losses of up to −15% (46, 47). In contrast, maize and sorghum show modest increases of up to +2% (45), attributable to differences in metabolic pathways (C3 vs. C4), along with nitrogen fixation in plants (45). Suggesting opportunities for climate-smart crop substitution strategies. Legumes appear more carbon-resilient, with minimal protein losses (< −5%), except for chickpeas (*Cicer arietinum*), which declined by −13.3% (45). Notably, common beans (*Phaseolus vulgaris*) demonstrate substantial gains in protein (∼ + 30%) and Fe (> + 50%) under water and temperature stress (48), highlighting breeding potential for stress-tolerant, nutrient-dense legume varieties.

Dry legumes exhibit greater reductions in mineral micronutrients than fresh forms, with an average of −16% difference in Zn and Fe losses observed between lentils (*Vicia lens*) and fava beans (*Vicia faba*) (49). Leafy vegetables, relative to their low sugar content, show the largest increase in soluble sugars (+36.2%), followed by fruits (*Solanum*), while root vegetables display the lowest gains under eCO_2_ (33, 34). Collectively, these patterns highlight the importance of targeted breeding and crop selection in enhancing resilience and nutritional value in future climate scenarios.

### 3.5 Quantifying the magnitude and direction of climate-driven nutrient alterations

**FIGURE 3:**
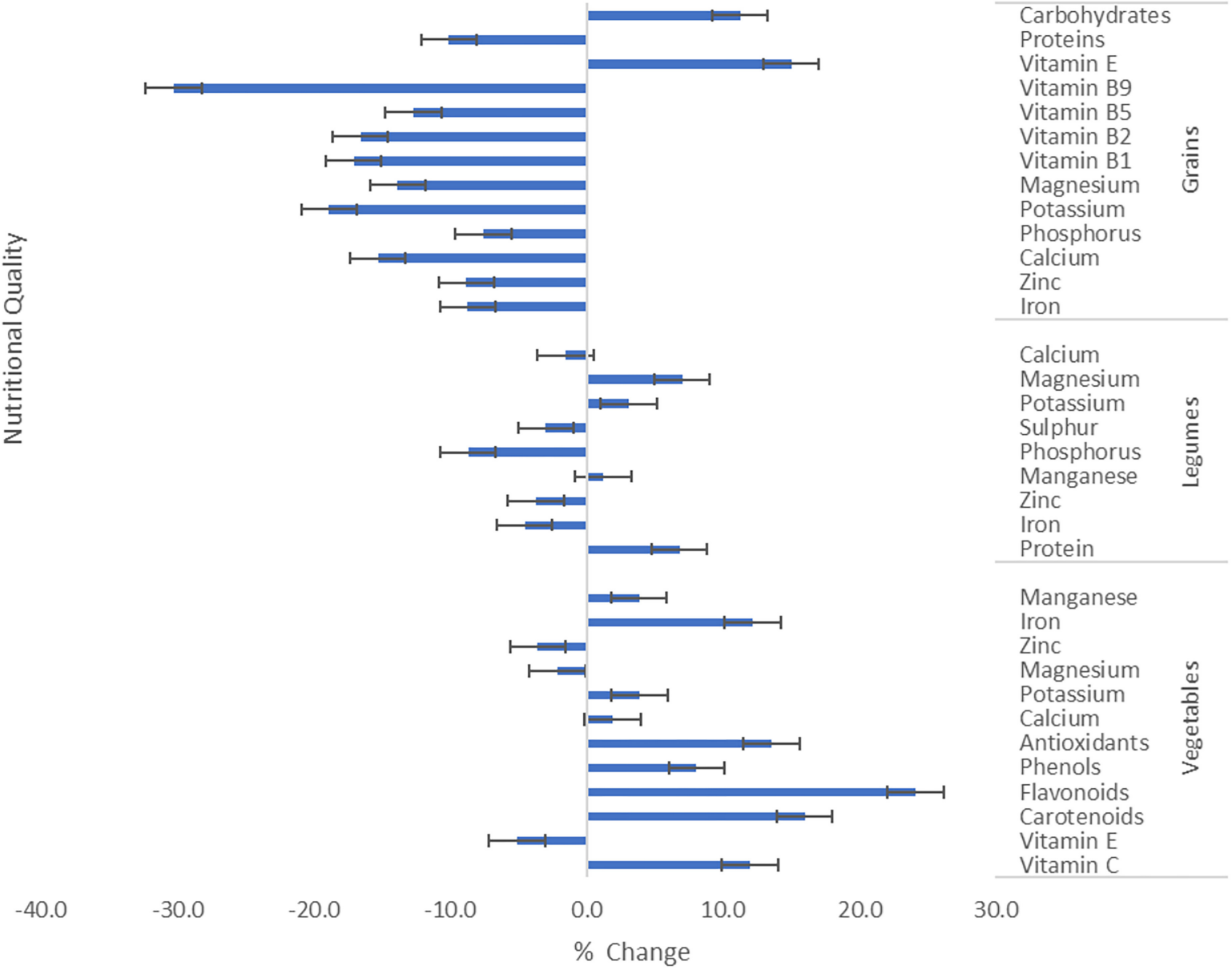
Experimental studies (*n* = 82) reported overall percentage changes in the nutritional quality of vegetable, legume, and grain crops exposed to cumulative climate stressors, including elevated CO_2_, temperature, and ozone, as well as water stress, salinity, and contamination.

**FIGURE 4:**
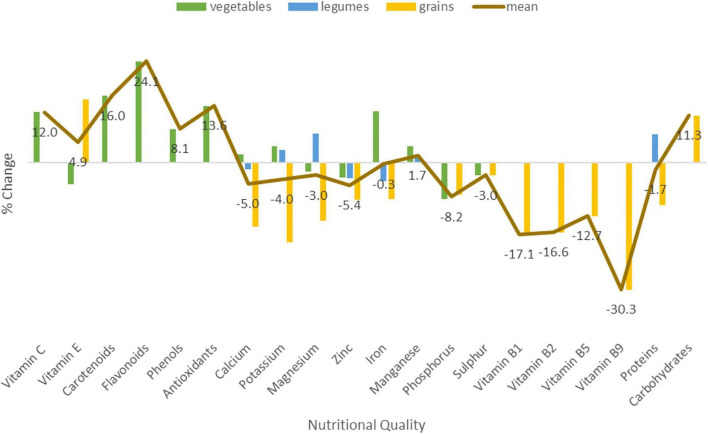
Meta-analysis studies (*n* = 9). Overall percentage changes in the nutritional quality of edible crops were derived from nine meta-analyses examining exposure to elevated CO_2_. Mean values for each nutrient parameter were calculated by averaging multiple data points: phytochemicals (*n* = 7), calcium (*n* = 3), potassium (*n* = 2), magnesium (*n* = 3), zinc (*n* = 3), iron (*n* = 3), manganese (*n* = 1), vitamin C (*n* = 2), protein (*n* = 8), and carbohydrate (*n* = 9).

**FIGURE 5:**
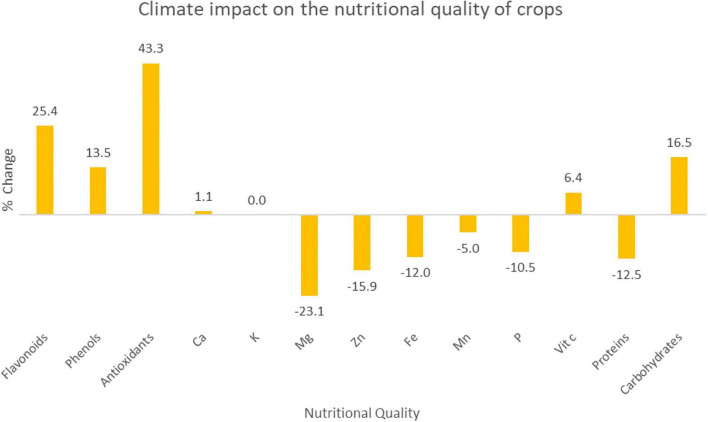
Overall percentage changes in dietary fiber, soluble sugars, and amino acids in grains exposed to elevated CO_2_. Soluble sugars increased by 13.2% (glucose), 14.2% (fructose), and 3.7% (sucrose). Amino acids decreased across categories: essential (–7.4%), semi-essential (–5.4%), non-essential (–8.6%), and child-essential (–8.1%).

Experimental studies (Supplementary Figures 1–3) consistently demonstrate reductions in crop nutrient content under climate stressors. Significant declines are observed in B vitamins (thiamine, riboflavin, niacin, and folate), protein, and essential minerals (Ca, K, Mg, Zn, Fe, P, and S), with an average loss of −3.0%. In contrast, increases were observed for vitamin C, vitamin E, carbohydrates, and antioxidants (Figure 3). Meta-analyses revealed similar trends, with greater declines in minerals (Figure 4), with an average decline of −8% in essential minerals, including Fe, Zn, and Ca. Meta-analysis and experimental studies revealed increases in soluble sugars (+12%) and declines in dietary fiber (−13.0%) and amino acids (−7.4%), respectively (Figure 5). Looking ahead, modeling studies project a global decline in nutrient availability of −4.4% for protein, −3.2% for Fe, and −3.0% for Zn. Collectively, these shifts compromise dietary quality and adequacy.

## 4 Discussion

Climate change is reshaping the nutritional composition of grains, legumes, and vegetables, generating new risks to public health. Alterations in crop macronutrients and micronutrients driven by elevated CO_2_, ozone (O_3_), rising temperatures, salinity, and water stress are (i) undermining diet quality and adequacy, (ii) predisposing populations to the multiple burdens of malnutrition, and (iii) challenging the resilience of food systems while necessitating a re-evaluation of dietary guidelines, practices, and policies.

### 4.1 Diet quality and adequacy

The review provides evidence of substantial declines in protein, vitamins, and essential minerals, coupled with increases in carbohydrates, thereby distorting the energy-nutrient balance of diets. In particular, the rise in simple sugars (glucose, fructose, and sucrose) alongside reductions in dietary fiber in rice and wheat reflects a shift in staple nutritional profiles from nutrient-dense to calorie-dense, effectively transforming them into “empty-calorie” foods (5). This transformation into “empty-calorie” foods diminishes dietary quality, implying heightened risks of obesity and metabolic disease, and potentially contributing to hidden hunger by meeting energy needs while failing to supply essential nutrients (5).

The noticeable decline in grain protein quality, particularly reductions in essential amino acids, poses increased risks for populations that rely on plant-based foods, whether due to cultural traditions, dietary transitions, or limited access to animal-source foods, making it harder for them to meet recommended nutrient intakes. In contrast, reduced protein density may be advantageous in populations where intake already exceeds recommended levels. This disparity underscores the importance of contextualizing dietary models, particularly when comparing low-income countries, where protein insufficiency is a pressing concern, with high-income countries, where excessive consumption is more prevalent.

Concurrent declines in crop essential minerals and folate may exacerbate global micronutrient deficiencies, contributing to impaired physical and cognitive development, greater susceptibility to infectious diseases in infants and young children, and reduced work productivity in adulthood (50–52). These challenges are further compounded by elevated levels of antinutritional compounds such as tannins, which diminish nutrient bioavailability (53) and possibly intensify risks of deficiencies among already vulnerable groups. Although certain climate-driven changes may yield nutritional benefits, for example, increased vitamin C enhances Fe absorption (54), and elevated antioxidant concentrations can protect against oxidative stress and chronic disease (55). These gains risk being offset by heavy metal contamination observed in rice and wheat under combined elevated CO_2_ and warming conditions (56). Safeguarding grain quality must be prioritized to counteract these adverse effects.

These climate-nutrient dynamics highlight troubling paradoxes: (i) nutrient losses may necessitate greater food consumption to meet dietary requirements, thereby increasing agricultural production and expanding the climate footprint; and (ii) although plant-based foods remain central to sustainable diets, climate change threatens their safety, nutritional quality, and adequacy. Populations dependent on undiversified plant-based diets are disproportionately affected (57, 58), posing a significant threat to nutrition security.

To address nutrient disparities, current evidence emphasises the importance of diversifying plant-based diets. Mixed farming of C3 and crops expands crop variety, thereby promoting dietary diversification, a key driver of improved nutritional outcomes for households, women, and children (59). The promotion of multi-grain products, particularly in complementary foods for infants and young children, can enhance the intake of essential amino acids such as cysteine and tyrosine, supporting healthy growth and development (60). Utilizing whole grains in cereal-based products may also mitigate climate-induced losses in dietary fiber. Incorporating legumes, C4 crops such as maize and sorghum, indigenous grains, fruits, and vegetables can help offset nutrient declines in C3 staples like rice and wheat, which are less resilient to environmental stress (45). Pairing legumes with grains improves protein quality (61, 62), while fresh legumes further strengthen nutrient adequacy. These strategies are particularly critical in regions such as sub-Saharan Africa, where cereals and pulses account for both caloric and micronutrient intake (14, 63, 64).

### 4.2 Malnutrition risks

The climate-driven nutrient penalties shift staple crops toward energy-dense but nutrient-poor profiles, hypothetically predisposing populations to the triple burden of undernutrition, obesity, and micronutrient deficiencies (2, 9, 65). Malnutrition remains the leading global cause of morbidity, mortality, and economic losses (66, 67). Overweight and obesity have become major risk factors for non-communicable diseases, now responsible for approximately 74% of global deaths (66). The observed increases in soluble sugar and reductions in dietary fiber in staple crops elevate the glycemic index, directly intensifying the risk and severity of type 2 diabetes mellitus (68).

Notable declines in essential micronutrients, such as Fe and Zn, are of public health significance (7). Billions of people are affected by micronutrient deficiencies, which carry lifelong consequences, including disability and premature death (69–74). Worldwide, one in two children aged 6–59 months and two in three women of reproductive age experience at least one micronutrient deficiency (6). The reductions in Fe and folate highlighted further exacerbate risks for these groups, who have heightened physiological demands for these nutrients during growth, reproduction, and early childhood (75). Women face greater vulnerability to anemia, reproductive dysfunction, and adverse birth outcomes (76–79). Inadequate Zn intake is strongly associated with childhood stunting (80, 81), while declines in essential amino acids impair both growth and cognitive development (60, 82). Moreover, insufficient protein intake contributes to intrauterine growth restriction and low birth weight (83), thereby perpetuating cycles of intergenerational malnutrition (84, 85).

Strengthening nutrition-sensitive climate adaptation strategies is essential. Key actions include training communities to adapt infant and young child feeding practices to climate-driven food availability, incorporating climate-resilient crops into school meals to enhance dietary diversity, aligning maternal nutrition counseling with climate challenges, raising public awareness of the links between nutrition and climate risks, and fostering collaboration across health, agriculture, and environment sectors to safeguard nutrition security. Embedding climate resilience within these interventions will ensure long-term sustainability and help break intergenerational cycles of malnutrition (12, 86).

### 4.3 Policy implications

Climate change is increasingly undermining diet quality and potentially human health by altering the nutrient content of staple crops, particularly protein and key micronutrients such as Fe and Zn. These pathways hypothetically increase risks of micronutrient deficiencies, anemia, stunting, and diet-related chronic diseases, particularly in low- and middle-income countries where food insecurity is already high (16). These findings have clear policy implications. Climate-related declines in nutrient density underscore the need for climate-responsive national dietary guidelines that reflect changing food composition and nutrient availability. They also strengthen the case for sustaining and expanding mandatory fortification policies, such as salt iodisation and wheat flour fortification, implemented in over 125 and 90 countries, respectively (87), and other global efforts such as the UN Decade of Action on Nutrition (88) and One Health-oriented food-system resilience plans. To buffer populations against climate-induced nutritional losses. A critical enabling action is updating food composition tables to reflect climate-sensitive nutrient changes, ensuring that dietary guidelines, nutritional surveillance, fortification design, labeling, and therapeutic feeding programmes are informed by accurate, climate-relevant data. Coordinated, multi-sectoral climate-smart policies are therefore essential to safeguard diet quality and population health under a changing climate.

Diversified diets and scaling up supplementation, especially for women of reproductive age and young children, are vital (89). Health programs such as antenatal and postnatal care platforms, community-based delivery of supplements, and targeted counseling by community health workers can be leveraged to improve uptake. Fortification efforts can be reinforced by focusing on centralized processing and distribution points such as commercial mills, industrial processors, and public procurement systems, supported by routine quality-control checks, micronutrient testing, and simple traceability systems. Retail markets, wholesalers, and school feeding or social protection programs provide additional environments for monitoring and ensuring fortified foods reach vulnerable populations. On the production side, agronomic biofortification and breeding of climate-resilient, nutrient-dense crops can be advanced across smallholder, rain-fed, irrigated, and climate-stressed environments through soil and foliar micronutrient application, improved and biofortified seed varieties, intercropping, and the use of indigenous climate-tolerant crops (90–92). Given the rising risks of heavy-metal contamination under climate stress, strengthening nutrient recycling, establishing monitoring protocols, and adopting greenhouse or field management practices that regulate CO_2_, temperature, and water quality are also essential to safeguard nutrient quality (10, 33, 93, 94).

The review highlights research gaps in important cereals in sub-Saharan Africa, such as sorghum, whose greater inclusion in diets could foster diverse, resilient, and healthy agri-food systems (95, 96). In addition, certain nutrients, such as B vitamins, remain understudied despite crops being a key source. Addressing these gaps requires diverse longitudinal studies to track nutrient shifts and strengthen surveillance systems for emerging deficiencies (30). Research and innovation remain pivotal in safeguarding nutrition under climate change by generating evidence, shaping solutions, and informing policy (27). Collectively, these findings point to the urgent need to integrate nutrition-sensitive adaptation into food and agriculture policies and to align agricultural incentives with climate resilience objectives, critical steps toward achieving Sustainable Development Goals 2 (zero hunger), 3 (good health and wellbeing), and 13 (climate action).

## 5 Strengths and limitations

The scoping review positions nutrition-sensitive climate change adaptation within the broader agriculture-health-environment nexus. The study resonates strongly with global frameworks such as the IPBES Nexus Report (97) and the planetary boundaries framework (98). Both of which warn that accelerating climate change, biogeochemical disruption, and food-system instability are jointly pushing human societies toward thresholds where nutrition, health, and ecosystem resilience can no longer be sustained without coordinated, cross-sectoral action. Drawing on studies published between 1950 and 2024, including experimental research, meta-analyses, and modeling, the review synthesizes evidence on the impacts of climate change on crop nutrition. Its wide geographic coverage strengthens the generalisability of the findings, while standardized search and data extraction procedures ensure methodological rigor.

Nonetheless, heterogeneity across studies, limited crop representation, and underreporting of confounding factors constrained quantitative synthesis, reinforcing the need for harmonized climate-nutrition reporting frameworks. Discrepant greenhouse and experiment findings highlight the supremacy of meta-analysis and the importance of standardization.

## 6 Conclusion

Climate change is altering the nutrient composition of crops, with the greatest losses observed in staples like wheat and rice, while water stress most severely affects vegetables. Although elevated CO_2_ and temperature combined show largely neutral effects, overall trends indicate increases in sugars, antioxidants, flavonoids, and vitamins C and E, alongside declines in protein, B vitamins, particularly folate, and key minerals including Fe, Zn, and Ca. Intensifying malnutrition risks through calorie-dense yet nutrient-poor diets. These findings highlight the urgent need to integrate climate adaptation and mitigation into food security and nutrition policies, including embedding sustainability criteria into national dietary guidelines. Research, innovation, and cross-sector collaboration remain pivotal to building food systems that are both climate-resilient and nutrition-sensitive.

## Data Availability

The original contributions presented in this study are included in this article/Supplementary material, further inquiries can be directed to the corresponding authors.
