# Supplementary material for: Safeguarding diet quality in a changing climate: a scoping review
Source: Front Nutr. 2026 May 28;13:1833758. doi: 10.3389/fnut.2026.1833758 (PMC13253482; doi:10.3389/fnut.2026.1833758)
Supplement: Supplementary file 1 [file Table_1.docx]

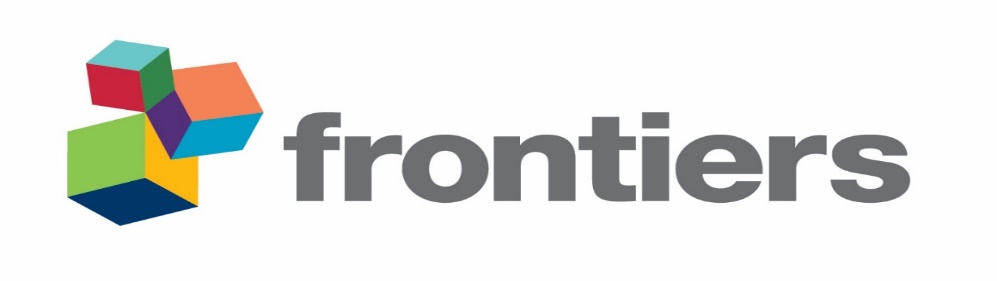


Supplementary Material

**Safeguarding Diet Quality in a Changing Climate: A Scoping Review**

Beaula Mutonhodza^1^,^6^*, Melody Ndemera^2^, Laurencia Govender^3^, Anouk Reuzé^4^, Mjabuliseni S. C. Ngidi^1^, Muthulisi Siwela^5^, Tafadzwanashe Mabhaudhi^1,4^

^1^Centre for Transformative Agricultural and Food Systems (CTAFS), School of Agriculture and Science, University of KwaZulu-Natal, Pietermaritzburg 3209, South Africa

^2^Department of Food Processing Technology, Harare Institute of Technology. P.O. Box BE277, Belvedere, Harare, Zimbabwe.

^3^Discipline of Dietetics and Human Nutrition, School of Health Sciences, University of KwaZulu-Natal, Private Bag X01, Scottsville 3209, Pietermaritzburg, South Africa

^4^Centre of Climate Change and Planetary Health, London School of Hygiene and Tropical Medicine (LSHTM), London WC1E 7HT, UK

^5^Discipline of Agricultural Management, School of Agriculture and Science, University of KwaZulu-Natal, Pietermaritzburg 3209, South Africa

^6^Department of Nutrition, Dietetics and Food Sciences, University of Zimbabwe. P.O. Box MP167, Mt Pleasant, Harare, Zimbabwe.

***Corresponding Author**:

Email: [MutonhodzaB@ukzn.ac.za](mailto:MutonhodzaB@ukzn.ac.za), [Tafadzwanashe.Mabhaudhi@lshtm.ac.uk](mailto:Tafadzwanashe.Mabhaudhi@lshtm.ac.uk)

**Table 1**: Experimental studies (n=42) reporting dual impacts of climate change on the nutritional quality of vegetables

| **Nutrient** | **Crop** | **Climate change exposure** | | | | | | **Change (%)** | **Experiment type** | **Reference** | **Country** |
| --- | --- | --- | --- | --- | --- | --- | --- | --- | --- | --- | --- |
|  |  | **eCO2** | **eTemp** | **eO3** | **Contaminated water** | **Water stress** | ***Salinity** |  |  |  |  |
| Vitamin C /Ascorbic acid | Tomato |  |  |  |  |  | x | -3.0 | Greenhouse | (1) | Germany |
|  |  |  |  |  |  |  | x | 0.7 | Field | (2) | India |
|  |  |  |  |  |  |  | x | -2.7 | Field | (3) | Austria |
|  |  |  |  |  |  |  | x | 1.8 | Field | (4) | China |
|  |  |  |  |  |  | x |  | 85.1 | Field | (5) | India |
|  |  |  |  |  |  | x |  | 59.5 | Field & Greenhouse | (6) | Bangladesh |
|  |  |  |  |  |  | x |  | 30.0 | Greenhouse | (7) | Spain |
|  |  |  |  |  |  | x |  | 20.2 | Greenhouse | (8) | China |
|  |  |  |  |  |  | x |  | 76.6 | Greenhouse | (9) | Germany |
|  |  |  |  |  |  | x |  | 9.72 | Field | (10) | Italy |
|  |  | x |  |  |  |  |  | 27.6 | Field | (11) | India |
|  | Lettuce | x |  |  |  |  |  | -4.9 | Greenhouse | (12) | Spain |
|  |  | x |  |  |  |  |  | 23.1 | Greenhouse | (13) | Spain |
|  |  |  |  |  |  | x |  | 4.4 | Greenhouse | (14) | United Kingdom (UK) |
|  |  |  |  |  | x |  |  | -6.6 | Greenhouse | (15) | China |
|  | Spinach | x |  |  |  |  |  | 11.7 | Greenhouse | (16) | China |
|  |  |  |  |  |  | x |  | 4.9 | Greenhouse | (14) | UK |
|  | Rocket |  |  |  |  |  | x | -0.02 | Greenhouse | (17) | United States (US) |
|  | Cress |  |  |  |  |  | x | -2.23 | Greenhouse | (17) | US |
|  | Celery |  |  |  |  | x |  | -2.2 | Greenhouse | (14) | UK |
|  | Cabbage |  |  |  |  | x |  | 1.7 | Greenhouse | (14) | UK |
|  | Parsley |  |  |  |  | x |  | 3.7 | Greenhouse | (14) | UK |
|  | Carrot |  |  |  |  | x |  | 4.7 | Greenhouse | (14) | UK |
|  | Onion |  |  |  |  | x |  | 12.9 | Greenhouse | (14) | UK |
|  | Celeriac |  |  |  |  | x |  | -3.7 | Greenhouse | (14) | UK |
|  | Olives |  |  |  |  | x |  | -7.2 | Field | (18) | Spain |
|  | Broccoli |  |  | x |  |  |  | 10.3 | Greenhouse | (19) | France |
|  | Pepper paprika |  |  |  |  | x |  | -20.5 | Field | (20) | Turkey |
| Vitamin E | Broccoli |  |  | x |  |  |  | -20.0 | Field | (21) | Belgium |
|  | Endive |  |  | x |  |  |  | -0.31 | Field | (21) | Belgium |
|  | Fennel |  |  | x |  |  |  | -0.31 | Field | (21) | Belgium |
|  | Kale |  |  |  |  | x |  | 48.1 | Greenhouse | (22) | Taiwan |
|  | Nightshade |  |  |  |  | x |  | -53.0 | Greenhouse | (22) | Taiwan |
| Carotenoids | Lettuce | x |  |  |  |  |  | -12.1 | Greenhouse | (12) | Spain |
|  |  | x |  |  |  |  |  | -8.2 | Greenhouse | (13) | Spain |
|  |  |  |  |  |  |  | x | 25.6 | Greenhouse | (23) | France |
|  | **Canola (young leaves) | x | x |  |  |  |  | -8.0 | Greenhouse | (24) | Canada |
|  |  | x | x |  |  | x |  | 17.0 | Greenhouse | (24) | Canada |
|  | Olive |  |  |  |  |  | x | 0.30 | Greenhouse | (25) | Tunisia |
|  | Pepper |  |  | x |  |  |  | 3.5 | Field | (26) | Saudi Arabia |
|  |  |  |  |  |  | x |  | -14.3 | Field | (20) | Turkey |
|  | Tomato |  |  |  |  | x |  | -6.2 | Field | (10) | Italy |
|  |  |  |  |  |  |  | x | 0.56 | Field | (27) | Italy |
|  |  |  |  |  |  |  | x | 1.3 | Field | (28) | Italy |
|  |  |  |  |  |  |  | x | -0.71 | Greenhouse | (1) | Germany |
|  |  | x |  |  |  |  |  | 11.2 | Greenhouse | (29) | Lithuania |
|  |  | x |  |  |  |  |  | -1.7 | Greenhouse | (11) | India |
|  |  |  |  |  |  | x |  | 304 | Field | (5) | India |
|  |  |  |  |  |  | x |  | 10.2 | Field | (30) | Hungary |
|  | Pea | x |  |  |  |  |  | 4.8 | Greenhouse | (29) | Lithuania |
|  | Sweet potato | x |  |  |  |  |  | -24.0 | Greenhouse | (31) | USA |
|  | Radish | x |  |  |  |  |  | -3.0 | Greenhouse | (29) | Lithuania |
|  | Kale |  |  |  |  | x |  | 19.1 | Greenhouse | (22) | Taiwan |
|  | Nightshade |  |  |  |  | x |  | 46.9 | Greenhouse | (22) | Taiwan |
|  | Spinach |  |  |  |  |  | x | 2.47 | Greenhouse | (32) | China |
|  |  | x |  | x |  |  |  | 70.0 | Greenhouse | (33) | India |
|  |  |  |  | x |  |  |  | -55.0 | Greenhouse | (33) | India |
| Flavonoids | Lettuce | x |  |  |  |  |  | 25.3 | Greenhouse | (12) | Spain |
|  |  | x |  |  |  |  |  | 4.5 | Greenhouse | (13) | Spain |
|  |  | x |  |  |  |  |  | 45 | Greenhouse | (34) | China |
|  | Tomato |  |  |  |  | x |  | 0.29 | Field | (10) | Italy |
|  |  |  |  |  |  | x |  | 99.4 | Field | (5) | India |
|  |  | x |  |  |  |  |  | -23.6 | Greenhouse | (11) | India |
|  | Olive |  |  |  |  | x |  | -0.9 | Field | (18) | Spain |
|  | Onion | x |  |  |  |  |  | 41 | Greenhouse | (35) | USA |
|  | Chinese cabbage | x |  |  |  |  |  | 70 | Greenhouse | (34) | China |
|  | Spinach | x |  |  |  |  |  | 1.9 | Greenhouse | (16) | China |
|  |  |  |  |  |  |  | x | 2.7 | Greenhouse | (32) | China |
| Phenols | Olive |  |  |  |  |  | x | 0.74 | Greenhouse | (25) | Tunisia |
|  |  |  |  |  |  | x |  | -0.24 | Field | (18) | Spain |
|  |  |  |  |  |  | x |  | -7.0 | Field | (36) | Italy |
|  | Tomato |  |  |  |  | x |  | -8.9 | Field | (10) | Italy |
|  |  |  |  |  |  | x |  | 55.3 | Field | (5) | India |
|  |  |  |  |  |  |  | x | -0.73 | Greenhouse | (1) | Germany |
|  | Rocket |  |  |  |  |  | x | -0.22 | Greenhouse | (17) | US |
|  | Lettuce | x |  |  |  |  |  | 11 | Greenhouse | (34) | China |
|  | Chinese cabbage | x |  |  |  |  |  | 30 | Greenhouse | (34) | China |
|  | Cress |  |  |  |  |  | x | 0.66 | Greenhouse | (17) | US |
| Antioxidants | Pepper |  |  | x |  |  |  | 2.0 | Field | (26) | Saudi Arabia |
|  | Tomato |  |  |  |  | x |  | -3.7 | Field | (10) | Italy |
|  |  |  |  |  |  | x |  | -31.4 | Field | (37) | Czech Republic |
|  |  |  |  |  |  | x |  | 24.3 | Field | (5) | India |
|  |  |  |  |  |  |  | x | 1.2 | Field | (38) | Italy |
|  | Lettuce | x |  |  |  |  |  | 89.2 | Greenhouse | (13) | Italy |
|  | Olive |  |  |  |  | x |  | -9.2 | Field | (18) | Spain |
|  | Broccoli |  |  | x |  |  |  | -9.3 | Field | (21) | Belgium |
|  | Spinach | x |  |  |  |  |  | 8.7 | Greenhouse | (16) | China |
|  |  |  |  |  |  |  | x | -0.27 | Greenhouse | (32) | China |
|  | Kale |  |  |  |  | x |  | 58.6 | Greenhouse | (22) | Taiwan |
|  | Nightshade |  |  |  |  | x |  | 48.2 | Greenhouse | (22) | Taiwan |
|  | Pepper |  |  |  |  | x |  | 3.62 | Field | (37) | Czech Republic |
|  | Leek |  |  |  |  | x |  | 7.8 | Field | (37) | Czech Republic |
| Calcium | Radish | x |  |  |  |  |  | -12.2 | Greenhouse | (39) | Germany |
|  | Olive |  |  |  |  | x |  | -24.4 | Field | (18) | Spain |
|  | Tomato |  |  |  |  |  | x | -0.03 | Field | (28) | Italy |
|  |  |  |  |  |  |  | x | -0.33 | Field | (27) | Italy |
|  |  |  |  |  | x |  |  | 8.1 | Field | (40) | Italy |
|  |  |  |  |  | x |  |  | -22.7 | Field | (40) | Italy |
|  | Cauliflower |  |  |  |  |  | x | 1.8 | Field | (38) | Italy |
|  | Broccoli |  |  |  |  |  | x | -6.7 | Field | (38) | Italy |
|  | Garlic |  |  |  |  |  | x | -0.98 | Field | (41) | US |
|  | Kale |  |  |  |  | x |  | 12.0 | Greenhouse | (22) | Taiwan |
|  | Nightshade |  |  |  |  | x |  | 48.8 | Greenhouse | (22) | Taiwan |
|  | Cabbage |  |  |  | x |  |  | 5.47 | Field | (42) | South Africa |
|  | Onion |  |  |  | x |  |  | 13.9 | Field | (42) | South Africa |
|  | Chard |  |  |  | x |  |  | 63.6 | Field | (42) | South Africa |
|  | Beetroot |  |  |  | x |  |  | 8.3 | Field | (42) | South Africa |
|  | Lettuce |  |  |  | x |  |  | -51.5 | Field | (42) | South Africa |
|  |  | x |  |  |  |  |  | 0.73 | Greenhouse | (43) | Spain |
|  |  | x |  |  |  |  |  | 5.8 | Greenhouse | (12) | Spain |
|  |  | x |  |  |  |  |  | 0.53 | Greenhouse | (19) | France |
|  | Carrot |  |  |  | x |  |  | -6.3 | Field | (42) | South Africa |
|  | Mustard | x |  |  |  |  |  | -4.2 | Field | (44) | India |
| Potassium (K) | Lettuce | x |  |  |  |  |  | 1.3 | Greenhouse | (12) | Spain |
|  |  | x |  |  |  |  |  | -4.6 | Greenhouse | (19) | France |
|  |  | x |  |  |  |  |  | 1.6 | Greenhouse | (43) | Spain |
|  |  |  |  |  | x |  |  | 28.6 | Field | (42) | South Africa |
|  | Radish | x |  |  |  |  |  | -5.1 | Greenhouse | (39) | Germany |
|  | Olive |  |  |  |  | x |  | -10.7 | Field | (18) | Spain |
|  | Tomato |  |  |  |  |  | x | -0.37 | Field | (28) | Italy |
|  |  |  |  |  |  |  | x | -0.5 | Field | (27) | Italy |
|  |  |  |  |  | x |  |  | 0.04 | Field | (45) | Italy |
|  | Cauliflower |  |  |  |  |  | x | -2.6 | Field | (38) | Italy |
|  | Broccoli |  |  |  |  |  | x | -1.4 | Field | (38) | Italy |
|  | Cabbage |  |  |  | x |  |  | -7.7 | Field | (42) | South Africa |
|  | Onion |  |  |  | x |  |  | -4.2 | Field | (42) | South Africa |
|  | Chard |  |  |  | x |  |  | 14.7 | Field | (42) | South Africa |
|  | Beetroot |  |  |  | x |  |  | 5.3 | Field | (42) | South Africa |
|  | Carrot |  |  |  | x |  |  | 47.7 | Field | (42) | South Africa |
| Magnesium (Mg) | Lettuce | x |  |  |  |  |  | 2.6 | Greenhouse | (12) | Spain |
|  |  | x |  |  |  |  |  | -8.2 | Greenhouse | (13) | Spain |
|  |  | x |  |  |  |  |  | 4.0 | Greenhouse | (19) | France |
|  |  |  |  |  | x |  |  | 12.5 | Field | (42) | South Africa |
|  | Radish | x |  |  |  |  |  | -14.8 | Greenhouse | (39) | Germany |
|  | Olive |  |  |  |  | x |  | -18.6 | Field | (18) | Spain |
|  | Tomato |  |  |  |  |  | x | -0.55 | Field | (28) | Italy |
|  |  |  |  |  | x |  |  | -2.5 | Field | (45) | Italy |
|  | Cauliflower |  |  |  |  |  | x | 1.0 | Field | (38) | Italy |
|  | Broccoli |  |  |  |  |  | x | -6.0 | Field | (38) | Italy |
|  | Cabbage |  |  |  | x |  |  | 12.5 | Field | (42) | South Africa |
|  | Beetroot |  |  |  | x |  |  | 16.7 | Field | (42) | South Africa |
|  | Carrot |  |  |  | x |  |  | -25.0 | Field | (42) | South Africa |
|  | Mustard | x |  |  |  |  |  | -3.7 | Field | (44) | India |
| Zinc (Zn) | Lettuce | x |  |  |  |  |  | -1.1 | Greenhouse | (12) | Spain |
|  |  | x |  |  |  |  |  | -2.3 | Greenhouse | (13) | Spain |
|  |  |  |  |  | x |  |  | -50.0 | Field | (42) | South Africa |
|  | Olive |  |  |  |  | x |  | -20.4 | Field | (18) | Spain |
|  | Cauliflower |  |  |  |  |  | x | -0.01 | Field | (38) | Italy |
|  | Broccoli |  |  |  |  |  | x | -8.7 | Field | (38) | Italy |
|  | Tomato | x |  |  |  |  |  | -8.8 | Greenhouse | (46) | Pakistan |
|  | Kale |  |  |  |  | x |  | 32.6 | Greenhouse | (22) | Taiwan |
|  | Nightshade |  |  |  |  | x |  | 9.9 | Greenhouse | (22) | Taiwan |
|  | Cabbage |  |  |  | x |  |  | 5.3 | Field | (42) | South Africa |
|  | Onion |  |  |  | x |  |  | 30.9 | Field | (42) | South Africa |
|  | Chard (spinach) |  |  |  | x |  |  | 32.7 | Field | (42) | South Africa |
|  | Turnip | x |  |  |  |  |  | -15.0 | Greenhouse | (46) | Pakistan |
|  | Radish | x |  |  |  |  |  | -17.0 | Greenhouse | (46) | Pakistan |
|  | Beetroot |  |  |  | x |  |  | 71.4 | Field | (42) | South Africa |
|  | Potato | x |  |  |  |  |  | -2.0 | Greenhouse | (47) | India |
|  |  | x |  | x |  |  |  | -15.0 | Greenhouse | (47) | India |
|  | Carrot |  |  |  | x |  |  | -30.0 | Field | (42) | South Africa |
|  |  | x |  |  |  |  |  | -17.0 | Greenhouse | (48) | Pakistan |
|  | Mustard | x |  |  |  |  |  | -4.2 | Field | (44) | India |
|  |  | x |  |  |  |  |  | -20.0 | Field | (49) | Japan |
|  | Spinach | x |  |  |  |  |  | -20.0 | Field | (49) | Japan |
|  | Cucumber | x |  |  |  |  |  | -34.0 | Greenhouse | (50) | China |
| Iron (Fe) | Lettuce | x |  |  |  |  |  | 243.7 | Greenhouse | (12) | Spain |
|  |  | x |  |  |  |  |  | 70.0 | Greenhouse | (19) | France |
|  |  | x |  |  |  |  |  | -13.3 | Greenhouse | (43) | Spain |
|  |  |  |  |  | x |  |  | 26.8 | Field | (42) | South Africa |
|  | Olive |  |  |  |  | x |  | -2.4 | Field | (18) | Spain |
|  | Tomato |  |  |  |  |  | x | -0.52 | Field | (28) | Italy |
|  |  | x |  |  |  |  |  | 3.4 | Greenhouse | (46) | Pakistan |
|  | Cauliflower |  |  |  |  |  | x | 6.6 | Field | (38) | Italy |
|  | Broccoli |  |  |  |  |  | x | -2.3 | Field | (38) | Italy |
|  | Spinach | x |  |  |  |  |  | -55.4 | Field | (51) | India |
|  |  | x |  |  |  |  |  | -30.0 |  | (52) | US |
|  | Kale |  |  |  |  | x |  | 9.9 | Greenhouse | (22) | Taiwan |
|  | Nightshade |  |  |  |  | x |  | 34.4 | Greenhouse | (22) | Taiwan |
|  | Cabbage |  |  |  | x |  |  | 166.5 | Field | (42) | South Africa |
|  | Onion |  |  |  | x |  |  | 53.8 | Field | (42) | South Africa |
|  | Chard |  |  |  | x |  |  | -30 | Field | (42) | South Africa |
|  | Potato | x |  |  |  |  |  | -25.0 | Greenhouse | (47) | India |
|  |  | x |  | x |  |  |  | -11.0 | Greenhouse | (47) | India |
|  | Beetroot |  |  |  | x |  |  | -40 | Field | (42) | South Africa |
|  | Radish | x |  |  |  |  |  | 3.0 | Greenhouse | (46) | Pakistan |
|  | Turnip | x |  |  |  |  |  | -19.0 | Greenhouse | (46) | Pakistan |
|  | Carrot |  |  |  | x |  |  | -30.0 | Field | (42) | South Africa |
|  |  | x |  |  |  |  |  | -8.0 | Greenhouse | (48) |  |
|  | Cucumber | x |  |  |  |  |  | -16.0 | Greenhouse | (53) | China |
|  |  | x |  |  |  |  |  | -31.0 | Greenhouse | (50) | China |
| Manganese (Mn) | Lettuce | x |  |  |  |  |  | 0.72 | Greenhouse | (12) | Spain |
|  |  |  |  |  | x |  |  | -13.4 | Field | (42) | South Africa |
|  | Olive |  |  |  |  | x |  | -9.5 | Field | (18) | Spain |
|  | Tomato |  |  |  |  |  | x | -0.69 | Field | (28) | Italy |
|  | Cauliflower |  |  |  |  |  | x | 6.0 | Field | (38) | Italy |
|  | Broccoli |  |  |  |  |  | x | 9.0 | Field | (38) | Italy |
|  | Cabbage |  |  |  | x |  |  | 30.0 | Field | (42) | South Africa |
|  | Onion |  |  |  | x |  |  | 58.6 | Field | (42) | South Africa |
|  | Chard |  |  |  | x |  |  | -3.1 | Field | (42) | South Africa |
|  | Beetroot |  |  |  | x |  |  | -32.2 | Field | (42) | South Africa |
|  | Carrot |  |  |  | x |  |  | -3.1 | Field | (42) | South Africa |

Notes: Results are reported for both field and greenhouse experiments, acknowledging possible concordance or discordance between these techniques (<https://doi.org/10.1111/j.1469-8137.2004.01224.x>) . Field and greenhouse findings may either reinforce or contradict one another. Values represent averages from studies that reported minimum and maximum ranges, and some studies assessed multiple climate exposures. The marked exposure corresponds to the recorded value. *Soil salinity was considered a climate change risk (<https://doi.org/10.1016/j.agwat.2024.109223>) **Canola leaves of young plants are commonly consumed as a leaf vegetable (<https://doi.org/10.1093/advances/nmab104>

**Table 2**: Experimental studies (n=19) reporting dual impacts of climate change on the nutritional quality of legumes

| **Nutrient** | **Crop** | **Climate change exposure** | | | | | | **Change (%)** | **Experiment type** | **Reference** | **Country** |
| --- | --- | --- | --- | --- | --- | --- | --- | --- | --- | --- | --- |
|  |  | **eCO2** | **eTemp** | **eO3** | **Contaminated water** | **Water Stress** | ***Salinity** |  |  |  |  |
| Protein | Common bean |  | x |  |  | x |  | 30.0 | Field | (54) | Malawi, Mozambique, Tanzania, Zambia, and Zimbabwe |
|  | Soya bean | x |  |  |  |  |  | -4.1 | Greenhouse | (55) | China |
|  |  | x |  |  |  |  |  | -8.0 | Field and greenhouse | (56) | USA, Australia |
|  | chickpea | x |  |  |  |  |  | 9.3 | Greenhouse | (57) | India |
| Iron (Fe) | Lentil (wet) | x |  |  |  |  |  | -4.0 | Greenhouse | (58) | Australia |
|  | Lentil (dry) | x |  |  |  |  |  | -20.0 | Greenhouse | (58) | Australia |
|  | Fava bean (wet) | x |  |  |  |  |  | -8.0 | Greenhouse | (58) | Australia |
|  | Fava bean (dry) | x |  |  |  |  |  | -22.0 | Greenhouse | (58) | Australia |
|  | Soybean | x |  |  |  |  |  | -10.0 | Greenhouse | (55) | China |
|  |  | x | x |  |  |  |  | -36.0 | Field & greenhouse | (56) | USA |
|  |  | x |  |  |  |  |  | -8.2 | Field | (59) | USA |
|  |  |  | x |  |  |  |  | 9.3 | Field | (59) | USA |
|  | Common bean |  |  |  |  | x |  | 3.5 | Field | (60) | Brazil |
|  |  |  | x |  |  | x |  | 50.0 | Field | (54) | Malawi, Mozambique, Tanzania, Zambia and Zimbabwe |
| Zinc (Zn) | Lentil (wet) | x |  |  |  |  |  | -6.0 | Greenhouse | (58) | Australia |
|  | Lentil (dry) | x |  |  |  |  |  | -24.0 | Greenhouse | (58) | Australia |
|  | Fava bean (wet) | x |  |  |  |  |  | -9.0 | Greenhouse | (58) | Australia |
|  | Fava bean (dry) | x |  |  |  |  |  | -25.0 | Greenhouse | (58) | Australia |
|  | Soybean | x |  |  |  |  |  | -4.0 | Greenhouse | (55) | China |
|  |  | x | x |  |  |  |  | -20.0 | Field & Greenhouse | (56) | USA |
|  |  | x |  |  |  |  |  | -8.9 | Field | (59) | USA |
|  |  |  | x |  |  |  |  | 4.9 | Field | (59) | USA |
|  | Chickpea | x |  |  |  |  |  | 38.5 | Greenhouse | (57) | India |
|  | Common Bean |  |  |  |  | x |  | 8.5 | Field | (60) | Brazil |
|  |  |  | x |  |  | x |  | 4.0 | Field | (54) | Malawi, Mozambique, Tanzania, Zambia and Zimbabwe |
| Manganese (Mn) | Soybean |  | x |  |  |  |  | 5.8 | Field | (59) | USA |
|  |  |  | x |  |  |  |  | -3.4 | Greenhouse | (61) | China |
| Phosphorus | Soybean |  | x |  |  |  |  | -10.9 | Field | (59) | US |
|  |  |  | x |  |  |  |  | -6.5 | Greenhouse | (61) | China |
| Sulphur (S) | Soybean |  | x |  |  |  |  | 3.6 | Field | (59) | US |
|  |  | x |  |  |  |  |  | -9.6 | Field | (59) | US |
| Potassium (K) | Soybean | x |  |  |  |  |  | 3.1 | Field | (59) | US |
| Magnesium (Mg) | Soybean |  | x |  |  |  |  | 7.0 | Field | (59) | US |
| Calcium (Ca) | Soybean | x |  |  |  |  |  | -5.5 | Field | (59) | US |
|  |  |  | x |  |  |  |  | -8.8 | Field | (59) | US |
|  |  |  | x |  |  |  |  | 9.6 | Greenhouse | (61) | China |

Notes: Results are reported for both field and greenhouse experiments, acknowledging that concordance or discordance between these approaches is possible (<https://doi.org/10.1111/j.1469-8137.2004.01224.x>) . Field and greenhouse findings may either reinforce or contradict one another. Values represent averages from studies that reported minimum and maximum ranges, with some studies assessing multiple climate exposures. The marked exposure corresponds to the recorded value. *Soil salinity was considered a climate change risk (<https://doi.org/10.1016/j.agwat.2024.109223>

**Table 3**: Experimental studies (n=21) reporting dual impacts of climate change on the nutritional quality of grains

| **Nutrient** | **Crop** | **Climate change exposure** | | | | | | **Change (%)** | **Experiment type** | **Reference** | **Country** |
| --- | --- | --- | --- | --- | --- | --- | --- | --- | --- | --- | --- |
|  |  | **eCO2** | **eTemp** | **eO3** | **Contaminated water** | **Water stress** | ***Salinity** |  |  |  |  |
| Iron (Fe) | Rice | x | x |  |  |  |  | -23.0 | Field | (62) | China |
|  |  | x | x |  |  |  |  | -18.5 | Greenhouse | (63) | India |
|  |  | x |  |  |  |  |  | -20.0 | Field | (64) | China |
|  |  | x |  |  |  |  |  | -16.0 | Greenhouse | (63) | India |
|  |  | x |  |  |  |  |  | -8.0 | Field | (65) | Japan, China |
|  |  | x |  |  |  |  |  | 14.0 | Greenhouse | (66) | Portugal |
|  |  | x |  |  |  |  |  | -18.0 | Greenhouse | (67) | Australia |
|  |  |  | x |  |  |  |  | 30.0 | Field | (64) | China |
|  | Wheat | x |  |  |  |  |  | -3.0 | Field | (64) | China |
|  |  | x |  |  |  |  |  | -20.0 | Greenhouse | (68) | Germany |
|  |  | x |  |  |  |  |  | -27.0 | Field | (69) | Italy |
|  |  | x |  |  |  |  |  | -6.0 | Field | (70) | Germany |
|  |  | x |  |  |  |  |  | -10.0 | Field | (71) | Australia |
|  |  | x |  |  |  |  |  | -15.0 | Field | (72) | China |
|  |  | x |  |  |  |  |  | -18.0 | Greenhouse | (73) | UK, Germany, Belgium, Denmark, Ireland, Netherlands |
|  |  | x |  |  |  |  |  | -26.5 | Greenhouse | (74) | UK, Germany, Belgium, Denmark, Ireland, Netherlands |
|  |  |  | x |  |  |  |  | 18.0 | Field | (64) | China |
|  |  | x |  |  |  |  |  | -5.1 | Field | (75) | Australia |
|  |  | x |  |  |  |  |  | -10.0 | Field | (76) | Australia |
|  | Maize |  | x |  |  |  |  | 7.3 | Greenhouse | (61) | China |
| Zinc (Zn) | Rice | x | x |  |  |  |  | 42.0 | Field | (62) | China |
|  |  | x | x |  |  |  |  | -16.5 | Greenhouse | (63) | India |
|  |  | x |  |  |  |  |  | -8.0 | Field | (64) | China |
|  |  | x |  |  |  |  |  | 8.0 | Greenhouse | (66) | Portugal |
|  |  | x |  |  |  |  |  | -5.0 | Field | (65) | China |
|  |  | x |  |  |  |  |  | -13.5 | Field | (63) | India |
|  |  | x |  |  |  |  |  | -15.0 | Greenhouse | (67) | Australia |
|  |  | x |  |  |  |  |  | -6.0 | Field | (49) | Japan |
|  |  |  | x |  |  |  |  | 7.0 | Field | (64) | China |
|  | Wheat | x |  |  |  |  |  | -14.0 | Field | (64) | China |
|  |  | x |  |  |  |  |  | -10.0 | Field | (77) | Australia |
|  |  | x |  |  |  |  |  | -20.0 | Greenhouse | (68) | Germany |
|  |  | x |  |  |  |  |  | -22.0 | Field | (69) | Italy |
|  |  | x |  |  |  |  |  | -10.0 | Greenhouse | (78) | Turkey |
|  |  | x |  |  |  |  |  | --5.8 | Field | (75) | Australia |
|  |  | x | x |  |  |  |  | -17.0 | Field | (76) | Australia |
|  |  | x | x |  |  | x |  | -22.0 | Field | (71) | Australia |
|  |  | x |  |  |  |  |  | -9.0 | Field | (72) | China |
|  |  | x |  |  |  |  |  | -33.0 | Greenhouse | (79) | China |
|  |  | x |  |  |  |  |  | -16.8 | Greenhouse | (74) | UK, Germany, Belgium, Denmark, Ireland, Netherlands |
|  |  |  | x |  |  |  |  | 4.0 | Field | (64) | China |
|  |  | x |  |  |  | x |  | -23.0 | Greenhouse | (78) | Turkey |
| Calcium (Ca) | Wheat | x |  |  |  |  |  | -19.5 | Field | (69) | Italy |
|  |  | x |  |  |  |  |  | -11.0 | Field | (76) | Australia |
|  |  | x |  |  |  |  |  | -6.5 | Field | (75) | Australia |
|  |  | x |  |  |  |  |  | -28.0 | Greenhouse | (68) | Germany |
|  |  |  | x |  |  |  |  | -11.6 | Greenhouse | (61) | China |
| Phosphorus (P) | Maize |  | x |  |  |  |  | 7.3 | Greenhouse | (61) | China |
|  |  | x |  |  |  |  |  | -19.0 | Greenhouse | (80) | India |
|  | Wheat | x |  |  |  |  |  | -11.0 | Field | (76) | Australia |
| Potassium (K) | Maize | x |  |  |  |  |  | -3.6 | Field | (75) | Australia |
|  |  | x |  |  |  |  |  | -5.0 | Greenhouse | (80) | India |
|  | Wheat | x |  |  |  |  |  | -22.4 | Field | (69) | Italy |
|  |  | x |  |  |  |  |  | -6.0 | Greenhouse | (68) | Germany |
|  |  | x |  |  |  |  |  | -57.5 | Field | (81) | Spain |
| Magnesium (Mg) | Wheat | x |  |  |  |  |  | -28.3 | Field | (69) | Italy |
|  |  | x |  |  |  |  |  | -15.3 | Field | (81) | Spain |
|  |  | x |  |  |  |  |  | -13.0 | Greenhouse | (68) | Germany |
|  |  | x |  |  |  |  |  | -7.0 | Field | (76) | Australia |
|  |  | x |  |  |  |  |  | -5.7 | Field | (75) | Australia |
| Vitamin B1 | Rice | x |  |  |  |  |  | -17.1 | Field | (65) | Japan, China |
| Vitamin B2 |  | x |  |  |  |  |  | -16.6 | Field | (65) | China |
| Vitamin B5 |  | x |  |  |  |  |  | -12.7 | Field | (65) | China |
| Vitamin B9 |  | x |  |  |  |  |  | -30.3 | Field | (65) | China |
| Vitamin E |  | x |  |  |  |  |  | 15.0 | Field | (65) | China |
| Proteins | Rice | x |  |  |  |  |  | -10.7 | Field | (82) | Germany |
|  |  | x |  |  |  |  |  | -10.3 | Field | (65) | China |
|  | Wheat | x | x |  |  | x |  | -12.7 | Field | (71) | Australia |
|  |  | x |  |  |  |  |  | -13.5 | Field | (76) | Australia |
|  |  | x |  |  |  |  |  | -6.1 | Field | (70) | Germany |
|  |  | x |  |  |  |  |  | -7.4 | Field | (82) | Germany |
| Essential amino acids |  | x |  |  |  |  |  | -7.4 |  |  |  |
| Semi essential amino acids |  | x |  |  |  |  |  | -5.4 |  |  |  |
| Non-essential amino acids |  | x |  |  |  |  |  | -8.6 |  |  |  |
| Essential for children |  | x |  |  |  |  |  | -8.1 |  |  |  |
| Serine (Sr) |  | x |  |  |  |  |  | -7.1 |  |  |  |
| Glutamic acid/  Glutamine (Glx) |  | x |  |  |  |  |  | -10.7 |  |  |  |
| Cysteine |  | x |  |  |  |  |  | --7.4 |  |  |  |
| Tyrosine |  | x |  |  |  |  |  | -8.9 |  |  |  |
| Histidine |  | x |  |  |  |  |  | -5.1 |  |  |  |
| Arginine |  | x |  |  |  |  |  | -5.6 |  |  |  |
| Tryptophan |  | x |  |  |  |  |  | -5.3 |  |  |  |
| Isoleucine |  | x |  |  |  |  |  | -8.7 |  |  |  |
| Carbohydrates | Wheat | x |  |  |  |  |  | 13.0 | Field | (82) | Germany |
| Starch |  | x |  |  |  |  |  | 8.0 | Field | (82) | Germany |
| Soluble sugars |  | x |  |  |  |  |  | 12.0 | Field | (82) | Germany |

Notes: Results are reported for both field and greenhouse experiments, recognizing that concordance or discordance between these approaches is possible (<https://doi.org/10.1111/j.1469-8137.2004.01224.x>). Field and greenhouse findings may either reinforce or contradict one another. Values represent averages from studies that reported minimum and maximum ranges, with some studies assessing multiple climate exposures. The marked exposure corresponds to the recorded value. Soil salinity was considered a climate change risk (<https://doi.org/10.1016/j.agwat.2024.109223>)

Table 4: Meta‑analysis (n=9) and modelling studies (n=2) reporting dual impacts of climate change on the nutritional quality of edible crops

| **Nutrient** | **Direction of change** | **% Change** | **Reference** |
| --- | --- | --- | --- |
| Meta-analysis | | | |
| Protein | ↓ | 11.0 | (83) |
| Overall mineral concentration | ↓ | 7.0 |
| Iron | ↑ | 17.0 | (84) |
| Vitamin C | ↑ | 3.2 |
| flavonoids | ↑ | 3.8 |
| Antioxidants | ↑ | 27.5 |
| Overall mineral concentration (except Mn) | ↓ | 8.0 | (85) |
| Protein | ↓ | 15.3 | (86) |
| Protein | ↓ | 23.0 | (87) |
| Carbohydrate | ↑ | 7.0 |
| Zinc | ↓ | 44.0 |
| Iron | ↓ | 44.0 |
| Calcium | ↓ | 20.0 |
| Phosphorus | ↓ | 16.0 |
| Sodium | ↓ | 18.0 |
| Potassium | ↑ | 10.0 |
| Magnesium | ↓ | 40.0 |
| Overall mineral concentration | ↓ | 4.0 | (88) |
| Proteins | ↓ | 10.0 | (89) |
| Carbohydrate | ↑ | 23.0 |
| Starch | ↑ | 50.0 |
| Soluble sugar | ↑ | 8.0 |
| Structural carbohydrate | ↓ | 13.0 |
| Phenolics | ↑ | 19.0 |
| Tannins | ↑ | 22.0 |
| Flavonoids | ↑ | 27.0 |
| Iron | ↓ | 5.0 | (90) |
| Zinc | ↓ | 13.0 |
| Potassium | ↓ | 10.0 |
| Magnesium | ↓ | 20.0 |
| Manganese | ↓ | 5.0 |
| Sodium | ↑ | 60.0 |
| Phosphorus | ↓ | 5.0 |
| Selenium | ↓ | 10.0 |
| Calcium | ↓ | 15.0 |
| Protein | ↓ | 9.5 | (91) |
| Magnesium | ↓ | 9.2 |
| Iron | ↓ | 16.0 |
| Zinc | ↓ | 9.4 |
| Glucose | ↑ | 13.2 |
| Fructose | ↑ | 14.2 |
| Sucrose | ↑ | 3.7 |
| Soluble sugar | ↑ | 17.5 |
| Total antioxidant | ↑ | 59.0 |
| Phenols | ↑ | 8.9 |
| Flavonoids | ↑ | 45.5 |
| Vitamin C | ↑ | 9.5 |
| Calcium | ↑ | 8.2 |
| Modelling | | | |
| Proteins | ↓ | 5.3 | (92) |
| Proteins | ↓ | 3.5 | (93) |
| Zinc | ↓ | 3.0 |
| Iron | ↓ | 3.2 |

Notes: Results reflect the impact of elevated carbon dioxide on C3 plants, which use the Calvin cycle for photosynthesis and account for approximately 85% of all plants, including wheat, rice, barley, potatoes, and spinach. The meta‑analyses considered both the direction and percentage change in edible crop nutrients. Earlier meta‑analyses, such as Jablonski et al. (2002) (<https://doi.org/10.1046/J.1469-8137.2002.00494.X>^) and Loladze (2002) (^<https://doi.org/10.1016/S0169-5347(02)02587-9>^), did not meet these inclusion criteria^.

**Table 5:** Studies (n=50) reporting neutral impacts of climate change on the nutritional quality of edible crops

| **Quality Parameter** | **eCO2** | **eTemp** | **Salinity** | **O3** | **Crops** |
| --- | --- | --- | --- | --- | --- |
| Carbohydrates | | | | | |
| Fructose | x |  |  |  | Cucumber (50,94) , potato (95,96) |
| Glucose | x |  |  |  | Broccoli (97), cucumber (50,94) , and potato (95,98) |
| Sucrose | x |  |  |  | Broccoli (97), potato (95,96,99), strawberry (100), sugar beet (101,102) and tomato (103–105) |
| Total soluble sugar | x |  |  |  | Broccoli (97), celtuce (16), hot pepper (106), lettuce (12,43,107) , potato(95,96) and tomato (108,109) |
| Non-reducing sugar | x |  |  |  | Tomato (46) and turnip (48) |
| Starch | x |  |  |  | Cucumber (50,94) , lettuce(12,13,110) , onion (111) and potato (95,96,99) |
| Dietary fibre | x |  |  |  | Cucumber (50), and tomato (46) |
| Proteins | | | | | |
| Total protein | x |  |  |  | Cabbage (112) , cucumber (50) and lettuce (12,13,110) , spinach (51), strawberry(100) and sweet pepper (113) |
| Soluble protein | x |  |  |  | Hot pepper (106) and lettuce (107,110) |
| Free amino acids | x |  |  |  | Chinese cabbage (114) and lettuce (115) |
| Antioxidants | | | | | |
| Ascorbic acid /Vitamin C | x |  |  |  | Hot pepper (106), lettuce (12,13,110) , palak (47), potato (96,99), spinach (116) and tomato (117) |
|  |  |  | x |  | Pepper (118), Spinach (119),Tomato(3) |
|  |  |  |  | x | Broccoli (120) |
| Total flavonoids | x |  |  |  | Lettuce (121,122) and chives and scallion (35) |
| Anthocyanins | x |  |  |  | Lettuce (12) |
| Total phenols | x |  |  |  | Cabbage (112) , Hongfengcai (123), lettuce (12,110), palak (33), scallion (124) and sweet pepper (113) |
|  |  |  | x |  | Pepper (118) |
| Glutathione | x |  |  |  | Hongfengcai (125) and lettuce (107,110) |
| Lycopene | x |  |  |  | Sweet pepper (113) and tomato (11,126–129) |
| Carotenoids | x |  |  |  | Lettuce (12,13,110) , palak (33) and tomato (11,129),sweet potato (31) |
|  | x | x |  |  | Canola (24) |
|  |  |  | x |  | Pepper (118) |
| β-carotene | x |  |  |  | Sweet pepper (113) and tomato (127) |
| Total antioxidant capacity | x |  |  |  | Hongfengcai (125), lettuce (122), scallion (124) and tomato (126) |
| Minerals | | | | | |
| Phosphorus (P) | x |  |  |  | Chinese cabbage (114), cucumber (50), lettuce (12,13,110), potato (99,130), sweet pepper (131) and tomato (132) |
| Potassium (K) | x |  |  |  | Carrot and radish (48), Chinese cabbage (114) , cucumber (50), sweet pepper (113,131) , lettuce (12,13,110), potato (133), sugar beet (102), and tomato (46,132) |
|  | x |  |  | x | Radish (39) |
|  |  |  | x |  | Spinach (119) |
| Calcium (Ca) | x |  |  |  | Chinese cabbage (114), cucumber (50), lettuce (43,107), sweet pepper (113,131) , potato (99,133), spinach (51) , and tomato (134) |
|  | x |  |  | x | Radish (39) |
|  |  |  | x |  | Spinach (119) |
|  | x |  |  | x | Mustard (135) |
| Magnesium (Mg) | x |  |  |  | Carrot (48), Chinese cabbage (114) , cucumber (50,94), lettuce (12,13,110), potato (99,130,136) and tomato (132,134) |
|  | x |  |  | x | Radish (39), Mustard (135) |
|  |  |  | x |  | Spinach (119) |
| Sulphur (S) | x |  |  |  | Broccoli (137), carrot and turnip (48), Chinese cabbage (114), cucumber (50), lettuce (43), potato (99), and tomato (46,134) |
| Iron (Fe) | x |  |  |  | Chinese cabbage (114), cucumber (50), fenugreek (138), lettuce (12,13,110), potato (99,130,133), rice (139,140), wheat (70,77,81), chickpea (57), rice (49) |
|  | x | x |  |  | Chinese cabbage (114) |
|  | x | x |  |  | *Soyabean (87,141), *wheat (87) |
|  |  |  | x |  | Spinach (119), Zucchini (142) |
|  | x |  |  | x | Mustard (135) |
| Manganese (Mn) | x |  |  |  | Chinese cabbage (114), cucumber (50), lettuce (107,110), potato (99,130,133), and sweet pepper (113,131) |
|  |  |  | x |  | Zucchini (142) |
| Copper (Cu) | x |  |  |  | Chinese cabbage (114), cucumber (50), sweet pepper (113,131), lettuce (12,13,110), and potato (133) |
|  |  |  | x |  | Zucchini (142) |
| Zinc (Zn) | x |  |  |  | Chinese cabbage (114), cucumber (50), lettuce (12,107,110), potato (99,130), rice (143), wheat (68,73) |
|  | x | x |  |  | Chinese cabbage (114) |
|  | x | x |  |  | *Soyabean (141) |
|  | x |  |  | x | rice (143), mustard (135) |
|  |  |  | x |  | Zucchini (142) |
| Sodium (Na) | x |  |  |  | Cucumber (50), lettuce (110), sweet pepper (113), and sugar beet (102) |
| Lead (Pb) | x |  |  |  | Carrot, radish, and turnip (48) |

Notes: Combined exposure to elevated temperature and carbon dioxide neutralizes the climate change impact on iron (Fe) and zinc (Zn)

References

1. Krauss S, Schnitzler WH, Grassmann J, Woitke M. The influence of different electrical conductivity values in a simplified recirculating soilless system on inner and outer fruit quality characteristics of tomato. J Agric Food Chem. 2006 Jan 25;54(2):441–8.

2. Nangare D, K GS, Satyendra K. Effect of blending fresh-saline water and discharge rate of drip on plant yield, water use efficiency (WUE) and quality of tomato in semi arid environment. African J Agric Res. 2013 Jul 18;8(27):3639–45.

3. Ullah SM, Gerzabek MH, Soja G. Effect of seawater and soil salinity on ion uptake, yield and quality of tomato (fruit). Bodenkultur. 1994;45(3):227–37.

4. Zhai Y, Yang Q, Hou M. The effects of saline water drip irrigation on tomato yield, quality, and blossom-end rot incidenceâ€"a 3a case study in the South of China. PLoS One. 2015 Nov 5;10(11).

5. Kumar PS, Singh Y, Nangare DD, Bhagat K, Kumar M, Taware PB, et al. Influence of growth stage specific water stress on the yield, physico-chemical quality and functional characteristics of tomato grown in shallow basaltic soils. Sci Hortic (Amsterdam). 2015 Dec 14;197:261–71.

6. Nahar K, Gretzmacher R. Effect of water stress on nutrient uptake, yield and quality of tomato (Lycopersicon esculentum Mill.) under subtropical conditions. Bodenkultur. 2002;53(1):45–51.

7. Sánchez-Rodríguez E, Rubio-Wilhelmi Mm, Cervilla LM, Blasco B, Rios JJ, Rosales MA, et al. Genotypic differences in some physiological parameters symptomatic for oxidative stress under moderate drought in tomato plants. Plant Sci [Internet]. 2010 Jan 1 [cited 2025 Jul 9];178(1):30–40. Available from: https://www.sciencedirect.com/science/article/abs/pii/S0168945209002647

8. Sun WH, Liu XY, Wang Y, Hua Q, Song XM, Gu Z, et al. Effect of water stress on yield and nutrition quality of tomato plant overexpressing StAPX. Biol Plant. 2014 Mar 1;58(1):99–104.

9. Veit-Köhler U, Krumbein A, Kosegarten H. Effect of different water supply on plant growth and fruit quality of Lycopersicon esculentum. J Plant Nutr Soil Sci [Internet]. 1999 Dec 1 [cited 2025 Jul 10];162(6):583–8. Available from: https://scispace.com/papers/effect-of-different-water-supply-on-plant-growth-and-fruit-2vq90adjou

10. Barbagallo RN, Di Silvestro I, Patanè C. Yield, physicochemical traits, antioxidant pattern, polyphenol oxidase activity and total visual quality of field-grown processing tomato cv. Brigade as affected by water stress in Mediterranean climate. J Sci Food Agric. 2013 Apr;93(6):1449–57.

11. Mamatha H, Srinivasa Rao NK, Laxman RH, Shivashankara KS, Bhatt RM, Pavithra KC. Impact of elevated CO2 on growth, physiology, yield, and quality of tomato (Lycopersicon esculentum Mill) cv. Arka Ashish. Photosynthetica. 2014 Nov 26;52(4):519–28.

12. Baslam M, Garmendia I, Goicoechea N. Elevated CO2 may impair the beneficial effect of arbuscular mycorrhizal fungi on the mineral and phytochemical quality of lettuce. Ann Appl Biol. 2012 Sep;161(2):180–91.

13. Pérez-López U, Miranda-Apodaca J, Muñoz-Rueda A, Mena-Petite A. Interacting effects of high light and elevated CO2 on the nutraceutical quality of two differently pigmented Lactuca sativa cultivars (Blonde of Paris Batavia and Oak Leaf). Sci Hortic (Amsterdam) [Internet]. 2015 Aug 6 [cited 2025 Jul 9];191:38–48. Available from: https://www.sciencedirect.com/science/article/abs/pii/S0304423815002435

14. Mogren LM, Beacham AM, Reade JPH, Monaghan JM. Moderate water stress prevents the postharvest decline of ascorbic acid in spinach (Spinacia oleracea L.) but not in spinach beet (Beta vulgaris L.). J Sci Food Agric [Internet]. 2016 Jul 1 [cited 2025 Jul 9];96(9):2976–80. Available from: https://pubmed.ncbi.nlm.nih.gov/26381599/

15. Meng H, Dong DM, Wang J, Yang KN, Tian L, Sun W, et al. Effects of simulated acid rain on main nutritional indicators of three leafy vegetables. Chem Res Chinese Univ. 2011;27(3):397–401.

16. Jin CW, Du ST, Zhang YS, Tang C, Lin XY. Atmospheric nitric oxide stimulates plant growth and improves the quality of spinach (Spinacia oleracea). Ann Appl Biol [Internet]. 2009 Aug 1 [cited 2025 Jul 10];155(1):113–20. Available from: https://scispace.com/papers/atmospheric-nitric-oxide-stimulates-plant-growth-and-1t400rjbjl

17. Hamilton JM, Fonseca JM. Effect of saline irrigation water on antioxidants in three hydroponically grown leafy vegetables: Diplotaxis tenuifolia, eruca sativa, and lepidium sativum. HortScience. 2010;45(4):546–52.

18. Cano-Lamadrid M, Hernández F, Corell M, Burló F, Legua P, Moriana A, et al. Antioxidant capacity, fatty acids profile, and descriptive sensory analysis of table olives as affected by deficit irrigation. J Sci Food Agric. 2017 Jan 30;97(2):444–51.

19. Chagvardieff P, d’Aletto T, André M. Specific effects of irradiance and CO2 concentration doublings on productivity and mineral content in lettuce. Adv Sp Res [Internet]. 1994 [cited 2025 Jul 9];14(11):269–75. Available from: https://pubmed.ncbi.nlm.nih.gov/11540192/

20. Kirnak H, Gökalp Z, Demır H, Kodal S, Yildirim E. Paprika Pepper Yield and Quality as Affected by Different Irrigation Levels. J Agric Sci [Internet]. 2016 Jan 1 [cited 2025 Jul 10];22(1):77–88. Available from: https://dergipark.org.tr/en/pub/ankutbd/issue/56546/786458

21. De Bock M, Guisez Y, Ceulemans R, Horemans N, Vandermeiren K. Impact of tropospheric ozone on food and feed quality of Brassica species. Comp Biochem Physiol Part A Mol Integr Physiol. 2009 Jun;153(2):S228.

22. Luoh JW, Begg CB, Symonds RC, Ledesma D, Yang R-Y, Luoh JW, et al. Nutritional Yield of African Indigenous Vegetables in Water-Deficient and Water-Sufficient Conditions. Food Nutr Sci [Internet]. 2014 Mar 31 [cited 2025 Jul 9];5(9):812–22. Available from: https://www.scirp.org/journal/paperinformation?paperid=44454

23. Borghesi E, González-Miret ML, Escudero-Gilete ML, Malorgio F, Heredia FJ, Meléndez-Martínez AJ. Effects of salinity stress on carotenoids, anthocyanins, and color of diverse tomato genotypes. J Agric Food Chem [Internet]. 2011 Nov 9 [cited 2025 Jul 9];59(21):11676–82. Available from: https://pubmed.ncbi.nlm.nih.gov/21923118/

24. Qaderi MM, Kurepin L V., Reid DM. Growth and physiological responses of canola (Brassica napus) to three components of global climate change: Temperature, carbon dioxide and drought. Physiol Plant. 2006 Dec;128(4):710–21.

25. Ahmed C Ben, Rouina B Ben, Sensoy S, Boukhriss M. Saline water irrigation effects on fruit development, quality, and phenolic Composition of virgin olive oils, cv. Chemlali. J Agric Food Chem. 2009 Apr 8;57(7):2803–11.

26. Al Sahli AA, Al-Muwayhi MA, Doaigey AR, Basalah MO, Ali HM, El-Zaidy M, et al. Effect of ozone and ascorbic acid on the anatomical, physiological and biochemical parameters of pepper [Capsicum frutescens L.). J Pure Appl Microbiol. 2013;7(SPEC. ISS. NOVEMB):159–68.

27. De Pascale S, Orsini F, Caputo R, Palermo MA, Barbieri G, Maggio A. Seasonal and multiannual effects of salinisation on tomato yield and fruit quality. Funct Plant Biol [Internet]. 2012 [cited 2025 Jul 9];39(8):689–98. Available from: https://pubmed.ncbi.nlm.nih.gov/32480820/

28. De Pascale S, Maggio A, Fogliano V, Ambrosino P, Ritieni A. Irrigation with saline water improves carotenoids content and antioxidant activity of tomato. J Hortic Sci Biotechnol [Internet]. 2001 [cited 2025 Jul 10];76(4):447–53. Available from: https://www.tandfonline.com/doi/abs/10.1080/14620316.2001.11511392

29. Juknys R., Duchovskis P., Sliesaravičius A., Šlepetys J., Januškaitiene I., Brazaityte A., et al. Response of different agricultural plants to elevated CO 2 and air temperature [Iogonekvairiuogonek žemės ūkio augaluogonek atsakas iogonek padidėjusius co 2 kiekiogonek ir oro temperatūraogonek]. Zemdirbyste [Internet]. 2011;98(3):259–66. Available from: http://www.scopus.com/inward/record.url?eid=2-s2.0-80053470728&partnerID=40&md5=0873e540c441d65a46ea64adf2bfe1f3

30. Pék Z, Szuvandzsiev P, Daood H, Neményi A, Helyes L. Effect of irrigation on yield parameters and antioxidant profiles of processing cherry tomato. Cent Eur J Biol [Internet]. 2014 Jan 1 [cited 2025 Jul 9];9(4):383–95. Available from: https://www.degruyterbrill.com/document/doi/10.2478/s11535-013-0279-5/html?srsltid=AfmBOopcbrFCAe2hSVq4UZB2zeCHy_9DJeptkk1uXFoizw-KDuGag05T

31. LU JY, BISWAS PK, PACE RD. Effect of Elevated CO2 Growth Conditions on the Nutritive Composition and Acceptability of Baked Sweet Potatoes. J Food Sci. 1986;51(2):358–9.

32. Xu C, Mou B. Responses of Spinach to Salinity and Nutrient Deficiency in Growth, Physiology, and Nutritional Value. J Am Soc Hortic Sci [Internet]. 2016 Jan 1 [cited 2025 Jul 10];141(1):12–21. Available from: https://journals.ashs.org/view/journals/jashs/141/1/article-p12.xml

33. Kumari S, Agrawal M, Tiwari S. Impact of elevated CO2 and elevated O3 on Beta vulgaris L.: Pigments, metabolites, antioxidants, growth and yield. Environ Pollut [Internet]. 2013 [cited 2025 Jul 9];174:279–88. Available from: https://pubmed.ncbi.nlm.nih.gov/23291007/

34. Fu Y, Shao L, Liu H, Li H, Zhao Z, Ye P, et al. Unexpected decrease in yield and antioxidants in vegetable at very high CO2 levels. Environ Chem Lett. 2015;13(4):473–9.

35. Thompson L, Peffley E, Green C, Paré P, Tissue D. Biomass, flavonol levels and sensory characteristics of allium cultivars grown hydroponically at ambient and elevated CO2. SAE Tech Pap. 2004;(724).

36. Palese AM, Nuzzo V, Favati F, Pietrafesa A, Celano G, Xiloyannis C. Effects of water deficit on the vegetative response, yield and oil quality of olive trees (Olea europaea L., cv Coratina) grown under intensive cultivation. Sci Hortic (Amsterdam). 2010 Jun 28;125(3):222–9.

37. Pokluda R, Petříková K, Kopta T, Jurica M, Jezdinský A, Vojtíšková J. The effect of irrigation on the economic and nutritional characteristics of selected vegetables. Acta Hortic. 2014 Jun 20;1038:231–8.

38. De Pascale S, Maggio A, Barbieri G. Soil salinization affects growth, yield and mineral composition of cauliflower and broccoli. Eur J Agron. 2005 Oct;23(3):254–64.

39. BARNES JD, PFIRRMANN T. The influence of CO2 and O3, singly and in combination, on gas exchange, growth and nutrient status of radish (Raphanus sativus L.). New Phytol [Internet]. 1992 [cited 2025 Jul 9];121(3):403–12. Available from: https://pubmed.ncbi.nlm.nih.gov/33874150/

40. Gatta G, Libutti A, Gagliardi A, Disciglio G, Beneduce L, D’Antuono M, et al. Effects of treated agro-industrial wastewater irrigation on tomato processing quality. Ital J Agron [Internet]. 2015;10(2):97–100. Available from: https://doi.org/10.4081/ija.2015.632

41. Francois LE. Yield and quality response of salt-stressed garlic. HortScience. 1994;29(11):1314–7.

42. Mzini LL, Winter K. Effects of irrigation water quality on vegetables Part 2: Chemical and nutritional content. South African J Plant Soil. 2015 Feb 27;32(1):33–7.

43. Pérez-López U, Miranda-Apodaca J, Lacuesta M, Mena-Petite A, Muñoz-Rueda A. Growth and nutritional quality improvement in two differently pigmented lettuce cultivars grown under elevated CO2 and/or salinity. Sci Hortic (Amsterdam). 2015 Nov 12;195:56–66.

44. Singh S, Bhatia A, Tomer R, Kumar V, Singh B, Singh SD. Synergistic action of tropospheric ozone and carbon dioxide on yield and nutritional quality of Indian mustard (Brassica juncea (L.) Czern.). Environ Monit Assess. 2013 Aug;185(8):6517–29.

45. Gatta G, Libutti A, Gagliardi A, Beneduce L, Brusetti L, Borruso L, et al. Treated agro-industrial wastewater irrigation of tomato crop: Effects on qualitative/quantitative characteristics of production and microbiological properties of the soil. Agric Water Manag [Internet]. 2015 Feb 1 [cited 2025 Jul 10];149:33–43. Available from: https://www.sciencedirect.com/science/article/pii/S0378377414003369

46. Khan I, Azam A, Mahmood A. The impact of enhanced atmospheric carbon dioxide on yield, proximate composition, elemental concentration, fatty acid and vitamin C contents of tomato (Lycopersicon esculentum). Environ Monit Assess. 2013 Jan;185(1):205–14.

47. Kumari S, Agrawal M. Growth, yield and quality attributes of a tropical potato variety (Solanum tuberosum L. cv Kufri chandramukhi) under ambient and elevated carbon dioxide and ozone and their interactions. Ecotoxicol Environ Saf. 2014 Mar;101(1):146–56.

48. Azam A, Khan I, Mahmood A, Hameed A. Yield, chemical composition and nutritional quality responses of carrot, radish and turnip to elevated atmospheric carbon dioxide. J Sci Food Agric. 2013;93(13):3237–44.

49. Ujiie K, Ishimaru K, Hirotsu N, Nagasaka S, Miyakoshi Y, Ota M, et al. How elevated CO2 affects our nutrition in rice, and how we can deal with it. PLoS One [Internet]. 2019 Mar 1 [cited 2025 Jul 9];14(3):e0212840. Available from: https://journals.plos.org/plosone/article?id=10.1371/journal.pone.0212840

50. Dong J, Xu Q, Gruda N, Chu W, Li X, Duan Z. Elevated and super-elevated CO2 differ in their interactive effects with nitrogen availability on fruit yield and quality of cucumber. J Sci Food Agric. 2018 Sep 1;98(12):4509–16.

51. Jain V, Pal M, Raj A, Khetarpal S. Photosynthesis and nutrient composition of spinach and fenugreek grown under elevated carbon dioxide concentration. Biol Plant. 2007 Sep;51(3):559–62.

52. Giri A, Armstrong B, Rajashekar CB. Elevated Carbon Dioxide Level Suppresses Nutritional Quality of Lettuce and Spinach. Am J Plant Sci. 2016;07(01):246–58.

53. DONG J long, LI X, Nazim G, DUAN Z qiang. Interactive effects of elevated carbon dioxide and nitrogen availability on fruit quality of cucumber (Cucumis sativus L.). J Integr Agric. 2018 Nov 1;17(11):2438–46.

54. Hummel M, Hallahan BF, Brychkova G, Ramirez-Villegas J, Guwela V, Chataika B, et al. Reduction in nutritional quality and growing area suitability of common bean under climate change induced drought stress in Africa. Sci Rep [Internet]. 2018 Dec 1 [cited 2025 Jul 9];8(1). Available from: https://pubmed.ncbi.nlm.nih.gov/30385766/

55. Li Y, Yu Z, Jin J, Zhang Q, Wang G, Liu C, et al. Impact of elevated CO2 on seed quality of soybean at the fresh edible and mature stages. Front Plant Sci. 2018;871.

56. Bellaloui N, Hu Y, Mengistu A, Abbas HK, Kassem MA, Tigabu M. Elevated Atmospheric Carbon Dioxide and Temperature Affect Seed Composition, Mineral Nutrition, and 15N and 13C Dynamics in Soybean Genotypes under Controlled Environments. Atlas J Plant Biol [Internet]. 2016 Jun 12 [cited 2025 Jul 9];56–65. Available from: https://journals.atlas-publishing.org/index.php/AJPB/article/view/114

57. Saha S, Chakraborty D, Sehgal VK, Pal M. Potential impact of rising atmospheric CO2 on quality of grains in chickpea (Cicer arietinum L.). Food Chem [Internet]. 2015 Nov 15 [cited 2025 Jul 9];187:431–6. Available from: https://pubmed.ncbi.nlm.nih.gov/25977047/

58. Parvin S, Uddin S, Tausz-Posch S, Armstrong R, Fitzgerald G, Tausz M. Grain mineral quality of dryland legumes as affected by elevated CO and drought: A FACE study on lentil (Lens culinaris) and faba bean (Vicia faba). Crop Pasture Sci [Internet]. 2019 Mar 14 [cited 2025 Jul 9];70(3):244–53. Available from: https://bioone.org/journals/crop-and-pasture-science/volume-70/issue-3/CP18421/Grain-mineral-quality-of-dryland-legumes-as-affected-by-elevated/10.1071/CP18421.full

59. Köhler IH, Huber SC, Bernacchi CJ, Baxter IR. Increased temperatures may safeguard the nutritional quality of crops under future elevated CO2 concentrations. Plant J [Internet]. 2019 Mar 1 [cited 2025 Jul 9];97(5):872–86. Available from: https://pubmed.ncbi.nlm.nih.gov/30447177/

60. Pereira HS, Del Peloso MJ, Bassinello PZ, Guimarães CM, Melo LC, Faria LC. Genetic variability for iron and zinc content in common bean lines and interaction with water availability. Genet Mol Res. 2014;13(3):6773–85.

61. Qiao Y, Miao S, Li Q, Jin J, Luo X, Tang C. Elevated CO 2 and temperature increase grain oil concentration but their impacts on grain yield differ between soybean and maize grown in a temperate region. Sci Total Environ. 2019;666(219):405–13.

62. Wei L, Wang W, Zhu J, Wang Z, Wang J, Li C, et al. Responses of rice qualitative characteristics to elevated carbon dioxide and higher temperature: implications for global nutrition. J Sci Food Agric [Internet]. 2021 Jul 1 [cited 2025 Jul 9];101(9):3854–61. Available from: https://pubmed.ncbi.nlm.nih.gov/33336371/

63. Chaturvedi AK, Bahuguna RN, Pal M, Shah D, Maurya S, Jagadish KSV. Elevated CO2 and heat stress interactions affect grain yield, quality and mineral nutrient composition in rice under field conditions. F Crop Res. 2017 May 1;206:149–57.

64. Wang J, Li L, Lam SK, Liu X, Pan G. Responses of wheat and rice grain mineral quality to elevated carbon dioxide and canopy warming. F Crop Res. 2020 Apr 1;249.

65. Zhu C, Kobayashi K, Loladze I, Zhu J, Jiang Q, Xu X, et al. Carbon dioxide (CO2) levels this century will alter the protein, micronutrients, and vitamin content of rice grains with potential health consequences for the poorest rice-dependent countries. Sci Adv. 2018;4(5):1–9.

66. Goufo P, Falco V, Brites C, Wessel DF, Kratz S, Rosa EAS, et al. Effect of elevated carbon dioxide concentration on rice quality: Nutritive value, color, milling, cooking, and eating qualities. Cereal Chem. 2014 Sep 1;91(5):513–21.

67. Seneweera SP, Conroy JP. Growth, grain yield and quality of rice (Oryza sativa L.) in response to elevated CO2 and phosphorus nutrition. Soil Sci Plant Nutr [Internet]. 1997 [cited 2025 Jul 9];43(SPEC. ISS.):1131–6. Available from: https://www.tandfonline.com/doi/pdf/10.1080/00380768.1997.11863730#:~:text=Page 2,stage of growth was reached.

68. Manderscheid R, Bender J, Jäger HJ, Weigel HJ. Effects of season long CO2 enrichment on cereals. II. Nutrient concentrations and grain quality. Agric Ecosyst Environ. 1995;54(3):175–85.

69. Beleggia R, Fragasso M, Miglietta F, Cattivelli L, Menga V, Nigro F, et al. Mineral composition of durum wheat grain and pasta under increasing atmospheric CO2 concentrations. Food Chem [Internet]. 2018 Mar 1 [cited 2025 Jul 7];242:53–61. Available from: https://pubmed.ncbi.nlm.nih.gov/29037725/

70. Högy P, Brunnbauer M, Koehler P, Schwadorf K, Breuer J, Franzaring J, et al. Grain quality characteristics of spring wheat (Triticum aestivum) as affected by free-air CO<SUB>2</SUB> enrichment. EnvEB [Internet]. 2013 Apr [cited 2025 Jul 9];88:11–8. Available from: https://ui.adsabs.harvard.edu/abs/2013EnvEB..88...11H/abstract

71. Fernando N, Panozzo J, Tausz M, Norton R, Fitzgerald G, Seneweera S. Rising atmospheric CO 2 concentration affects mineral nutrient and protein concentration of wheat grain. Food Chem. 2012 Aug 15;133(4):1307–11.

72. Wu DX, Wang GX, Bai YF, Liao JX. Effects of elevated CO 2 concentration on growth, water use, yield and grain quality of wheat under two soil water levels. Agric Ecosyst Environ. 2004 Dec;104(3):493–507.

73. Fangmeier A, Grüters U, Högy P, Vermehren B, Jäger HJ. Effects of elevated CO2, nitrogen supply and tropospheric ozone on spring wheat - II. Nutrients (N, P, K, S, Ca, Mg, Fe, Mn, Zn). Environ Pollut [Internet]. 1997 [cited 2025 Jul 9];96(1):43–59. Available from: https://pubmed.ncbi.nlm.nih.gov/15093431/

74. Fangmeier A, De Temmerman L, Mortensen L, Kemp K, Burke J, Mitchell R, et al. Effects on nutrients and on grain quality in spring wheat crops grown under elevated CO2 concentrations and stress conditions in the European, multiple-site experiment “ESPACE-wheat.” Eur J Agron [Internet]. 1999 Apr [cited 2025 Jul 9];10(3–4):215–29. Available from: https://research.wur.nl/en/publications/effects-on-nutrients-and-on-grain-quality-in-spring-wheat-crops-g

75. Fernando N, Panozzo J, Tausz M, Norton RM, Neumann N, Fitzgerald GJ, et al. Elevated CO2 alters grain quality of two bread wheat cultivars grown under different environmental conditions. Agric Ecosyst Environ. 2014 Mar 1;185:24–33.

76. Fernando N, Panozzo J, Tausz M, Norton RM, Fitzgerald GJ, Myers S, et al. Wheat grain quality under increasing atmospheric CO 2 concentrations in a semi-arid cropping system. J Cereal Sci. 2012 Nov;56(3):684–90.

77. Jin J, Armstrong R, Tang C. Impact of elevated CO2 on grain nutrient concentration varies with crops and soils – A long-term FACE study. Sci Total Environ [Internet]. 2019 Feb 15 [cited 2025 Jul 7];651:2641–7. Available from: https://www.sciencedirect.com/science/article/abs/pii/S0048969718340531

78. Asif M, Yilmaz O, Ozturk L. Elevated carbon dioxide ameliorates the effect of Zn deficiency and terminal drought on wheat grain yield but compromises nutritional quality. Plant Soil. 2017 Feb 1;411(1–2):57–67.

79. Erbs M, Manderscheid R, Jansen G, Seddig S, Pacholski A, Weigel HJ. Effects of free-air CO2 enrichment and nitrogen supply on grain quality parameters and elemental composition of wheat and barley grown in a crop rotation. Agric Ecosyst Environ. 2010 Feb 15;136(1–2):59–68.

80. Abebe A, Pathak H, Singh SD, Bhatia A, Harit RC, Kumar V. Growth, yield and quality of maize with elevated atmospheric carbon dioxide and temperature in north-west India. Agric Ecosyst Environ. 2016 Feb 15;218:66–72.

81. Sánchez De La Puente L, Pérez Pérez P, Martínez-Carrasco R, Morcuende Morcuende R, Martín Del Molino IM. Action of elevated C02 and high temperatures on the mineral chemical composition of two varieties of wheat. Agrochimica. 2000;44(5–6):221–30.

82. Högy P, Wieser H, Köhler P, Schwadorf K, Breuer J, Franzaring J, et al. Effects of elevated CO2 on grain yield and quality of wheat: Results from a 3-year free-air CO2 enrichment experiment. Plant Biol. 2009 Nov;11(SUPPL.1):60–9.

83. Myers SS, Smith MR, Guth S, Golden CD, Vaitla B, Mueller ND, et al. Climate Change and Global Food Systems: Potential Impacts on Food Security and Undernutrition. Annu Rev Public Health. 2017;38:259–77.

84. Scheelbeek PFD, Bird FA, Tuomisto HL, Green R, Harris FB, Joy EJM, et al. Effect of environmental changes on vegetable and legume yields and nutritional quality. Proc Natl Acad Sci U S A [Internet]. 2018 Jun 26 [cited 2025 Jul 8];115(26):6804–9. Available from: /doi/pdf/10.1073/pnas.1800442115?download=true

85. Loladze I. Hidden shift of the ionome of plants exposed to elevated CO2 depletes minerals at the base of human nutrition. Elife. 2014 May 7;2014(3).

86. Taub DR, Miller B, Allen H. Effects of elevated CO2 on the protein concentration of food crops: A meta-analysis. Glob Chang Biol. 2008;14(3):565–75.

87. Mariem S Ben, Gámez AL, Larraya L, Fuertes-Mendizabal T, Cañameras N, Araus JL, et al. Assessing the evolution of wheat grain traits during the last 166 years using archived samples. Sci Rep [Internet]. 2020;10(1):1–14. Available from: https://doi.org/10.1038/s41598-020-78504-x

88. Broberg MC, Högy P, Pleijel H. CO2-induced changes in wheat grain composition: Meta-analysis and response functions. Agronomy. 2017 Apr 25;7(2).

89. Robinson EA, Ryan GD, Newman JA. A meta-analytical review of the effects of elevated CO 2 on plant-arthropod interactions highlights the importance of interacting environmental and biological variables. New Phytol. 2012;194(2):321–36.

90. Mcgrath JM, Lobell DB. Reduction of transpiration and altered nutrient allocation contribute to nutrient decline of crops grown in elevated CO2 concentrations. Plant, Cell Environ [Internet]. 2013 Mar 1 [cited 2025 Jul 24];36(3):697–705. Available from: /doi/pdf/10.1111/pce.12007

91. Dong J, Gruda N, Lam SK, Li X, Duan Z. Effects of elevated CO2 on nutritional quality of vegetables: A review. Front Plant Sci. 2018 Aug 15;9.

92. Medek DE, Schwartz J, Myers SS. Estimated effects of future atmospheric co2 concentrations on protein intake and the risk of protein deficiency by country and region. Environ Health Perspect. 2017;125(8).

93. Beach RH, Sulser TB, Crimmins A, Cenacchi N, Cole J, Fukagawa NK, et al. Combining the effects of increased atmospheric carbon dioxide on protein, iron, and zinc availability and projected climate change on global diets: a modelling study. Lancet Planet Heal [Internet]. 2019;3(7):e307–17. Available from: http://dx.doi.org/10.1016/S2542-5196(19)30094-4

94. Tang, Y., Dong, J., Li, X., Gruda, N. and Duan Z. Interactive effects of elevated carbon dioxide and nitrogen availability on fruit quality of cucumber (Cucumis sativus L.). J Integr Agric.

95. Donnelly A, Lawson T, Craigon J, Black CR, Colls JJ, Landon G. Effects of elevated CO2 and O3 on tuber quality in potato (Solanum tuberosum L.). Agric Ecosyst Environ [Internet]. 2001 Dec 1 [cited 2025 Jul 24];87(3):273–85. Available from: https://www.sciencedirect.com/science/article/abs/pii/S016788090100144X

96. Vorne V, Ojanperä K, De Temmerman L, Bindi M, Högy P, Jones MB, et al. Effects of elevated carbon dioxide and ozone on potato tuber quality in the European multiple-site experiment “CHIP-project.” Eur J Agron. 2002 Nov;17(4):369–81.

97. Krumbein A, Kläring HP, Schonhof I, Schreiner M. Atmospheric carbon dioxide changes photochemical activity, soluble sugars and volatile levels in broccoli brassica oleracea var. italica. J Agric Food Chem. 2010;58(6):3747–52.

98. Vorne V, Ojanperä K, De Temmerman L, Bindi M, Högy P, Jones MB, et al. Effects of elevated carbon dioxide and ozone on potato tuber quality in the European multiple-site experiment “CHIP-project.” Eur J Agron. 2002;17(4):369–81.

99. Högy P, Fangmeier A. Atmospheric CO2 enrichment affects potatoes: 2. Tuber quality traits. Eur J Agron [Internet]. 2009 Feb 1 [cited 2025 Jul 9];30(2):85–94. Available from: https://www.sciencedirect.com/science/article/abs/pii/S1161030108000877

100. Sun P, Mantri N, Lou H, Hu Y, Sun D, Zhu Y, et al. Effects of elevated CO2 and temperature on yield and fruit quality of strawberry (Fragaria × ananassa Duch.) at two levels of nitrogen application. PLoS One. 2012;7(7).

101. Demmers-Derks H, Mitchell RAC, Mitchell VJ, Lawlor DW. Response of sugar beet (Beta vulgaris L.) yield and biochemical composition to elevated CO2 and temperature at two nitrogen applications. Plant, Cell Environ. 1998 Aug;21(8):829–36.

102. Manderscheid R, Pacholski A, Weigel HJ. Effect of free air carbon dioxide enrichment combined with two nitrogen levels on growth, yield and yield quality of sugar beet: Evidence for a sink limitation of beet growth under elevated CO2. Eur J Agron. 2010;32(3):228–39.

103. Behboudian MH, Tod C. Postharvest Attributes of `Virosa’ Tomato Fruit Produced in an Enriched Carbon Dioxide Environment. HortScience. 2019;30(3):490–1.

104. ISLAM MS, MATSUI T, YOSHIDA Y. Effects of Carbon Dioxide Enrichment on Acid Invertase and Sugar Concentration in Developing Tomato Fruit. Environ Control Biol. 1994;32(4):245–51.

105. Islam MS, Matsui T, Yoshida Y. Effect of carbon dioxide enrichment on physico-chemical and enzymatic changes in tomato fruits at various stages of maturity. Sci Hortic (Amsterdam). 1996;65(2–3):137–49.

106. Li XJ, Kang SZ, Li FS, Zhang XT, Huo ZL, Ding RS, et al. Light supplement and carbon dioxide enrichment affect yield and quality of off-season pepper. Agron J. 2017;109(5):2107–18.

107. Pérez-López U, Miranda-Apodaca J, Muñoz-Rueda A, Mena-Petite A. Interacting effects of high light and elevated CO2 on the nutraceutical quality of two differently pigmented Lactuca sativa cultivars (Blonde of Paris Batavia and Oak Leaf). Sci Hortic (Amsterdam). 2015;191:38–48.

108. Li JH, Sagi M, Gale J, Volokita M, Novoplansky A. Response of tomato plants to saline water as affected by carbon dioxide supplementation. I. Growth, yield and fruit quality. J Hortic Sci Biotechnol. 1999;74(2):232–7.

109. Wei Z, Du T, Li X, Fang L, Liu F. Interactive effects of elevated CO2 and N fertilization on yield and quality of tomato grown under reduced irrigation regimes. Front Plant Sci. 2018;9(March):1–10.

110. Pérez-López U, Miranda-Apodaca J, Lacuesta M, Mena-Petite A, Muñoz-Rueda A. Growth and nutritional quality improvement in two differently pigmented lettuce cultivars grown under elevated CO2 and/or salinity. Sci Hortic (Amsterdam). 2015;195:56–66.

111. Bettoni MM, Mogor ÁF, Pauletti V, Goicoechea N. The interaction between mycorrhizal inoculation, humic acids supply and elevated atmospheric CO2 increases energetic and antioxidant properties and sweetness of yellow onion. Hortic Environ Biotechnol. 2017;58(5):432–40.

112. Reddy GVP, Tossavainen P, Nerg AM, Holopainen JK. Elevated atmospheric CO2 affects the chemical quality of Brassica plants and the growth rate of the specialist, Plutella xylostella, but not the generalist, Spodoptera littoralis. J Agric Food Chem. 2004;52(13):4185–91.

113. Pinero MC, Otálora G, Porras ME, Sánchez-Guerrero MC, Lorenzo P, Medrano E, et al. The form in which nitrogen is supplied affects the polyamines, amino acids, and mineral composition of sweet pepper fruit under an elevated CO2 concentration. J Agric Food Chem. 2017;65(4):711–7.

114. Reich M, van den Meerakker AN, Parmar S, Hawkesford MJ, De Kok LJ. Temperature determines size and direction of effects of elevated CO2 and nitrogen form on yield quantity and quality of Chinese cabbage. Plant Biol [Internet]. 2016 Jan 1 [cited 2025 Jul 9];18:63–75. Available from: https://pubmed.ncbi.nlm.nih.gov/26390257/

115. Miyagi A, Uchimiya H, Kawai-Yamada M. Synergistic effects of light quality, carbon dioxide and nutrients on metabolite compositions of head lettuce under artificial growth conditions mimicking a plant factory. Food Chem [Internet]. 2017 Mar 1 [cited 2025 Jul 24];218:561–8. Available from: https://www.sciencedirect.com/science/article/abs/pii/S0308814616314972?via%3Dihub

116. Seo Y, Ide K, Kitahata N, Kuchitsu K, Dowaki K. Environmental impact and nutritional improvement of elevated CO2 treatment: A case study of spinach production. Sustain. 2017;9(10).

117. Ozcelik N, Akilh M. Effects of CO2 enrichment on vegetative growth, yield and quality of greenhouse - Grown tomatoes in soil and soilless cultures. Vol. 491, Acta Horticulturae. 1999. p. 155–60.

118. Navarro JM, Flores P, Garrido C, Martinez V. Changes in the contents of antioxidant compounds in pepper fruits at different ripening stages, as affected by salinity. Food Chem [Internet]. 2006 May 1 [cited 2025 Jul 9];96(1):66–73. Available from: https://www.sciencedirect.com/science/article/abs/pii/S0308814605001585

119. Shimomachi T, Kawahara Y, Kobashigawa C, Omoda E, Hamabe K, Tamaya K. Effect of residual salinity on spinach growth and nutrient contents in polder soil. Acta Hortic. 2008;797:419–24.

120. Vandermeiren K, De Bock M, Horemans N, Guisez Y, Ceulemans R, De Temmerman L. Ozone effects on yield quality of spring oilseed rape and broccoli. Atmos Environ [Internet]. 2012 Feb 1 [cited 2025 Jul 10];47:76–83. Available from: https://www.sciencedirect.com/science/article/abs/pii/S1352231011012210

121. Becker C, Kläring HP. CO2 enrichment can produce high red leaf lettuce yield while increasing most flavonoid glycoside and some caffeic acid derivative concentrations. Food Chem [Internet]. 2016 May 15 [cited 2025 Jul 24];199:736–45. Available from: https://www.sciencedirect.com/science/article/pii/S030881461530337X?via%3Dihub

122. Pérez-López U, Sgherri C, Miranda-Apodaca J, Micaelli F, Lacuesta M, Mena-Petite A, et al. Concentration of phenolic compounds is increased in lettuce grown under high light intensity and elevated CO2. Plant Physiol Biochem [Internet]. 2018;123(October 2017):233–41. Available from: https://doi.org/10.1016/j.plaphy.2017.12.010

123. Ren J, Guo SS, Xin XL, Chen L. Changes in volatile constituents and phenols from Gynura bicolor DC grown in elevated CO2 and LED lighting. Sci Hortic (Amsterdam) [Internet]. 2014;175:243–50. Available from: http://dx.doi.org/10.1016/j.scienta.2014.06.023

124. Levine LH, Paré PW. Antioxidant capacity reduced in scallions grown under elevated CO 2 independent of assayed light intensity. Adv Sp Res. 2009;44(8):887–94.

125. Wang M, Liu H, Dong C, Fu Y. Elevated CO2 enhances photosynthetic efficiency, ion uptake and antioxidant activity of Gynura bicolor DC. grown in a porous-tube nutrient delivery system under simulated microgravity. Plant Biol. 2016;18(3):391–9.

126. Helyes L, Lugasi A, Neményi A, Pék Z. The simultaneous effect of elevated co2-level and nitrogen-supply on the fruit components of tomato. Acta Aliment. 2012;41(2):265–71.

127. Krumbein A, Schwarz D, Kläring HP. Effects of environmental factors on carotenoid content in tomato (Lycopersicon esculentum (L.) Mill.) grown in a greenhouse. J Appl Bot Food Qual. 2006;80(2):160–4.

128. Li F, Wang J, Chen Y, Zou Z, Wang X, Yue M. Combined effects of enhanced ultraviolet-B radiation and doubled CO 2 concentration on growth, fruit quality and yield of tomato in winter plastic greenhouse. Front Biol China. 2007;2(4):414–8.

129. Zhang Z, Liu L, Zhang M, Zhang Y, Wang Q. Effect of carbon dioxide enrichment on health-promoting compounds and organoleptic properties of tomato fruits grown in greenhouse. Food Chem [Internet]. 2014;153:157–63. Available from: http://dx.doi.org/10.1016/j.foodchem.2013.12.052

130. Fangmeier A, De Temmerman L, Black C, Persson K, Vorne V. Effects of elevated CO2 and/or ozone on nutrient concentrations and nutrient uptake of potatoes. Eur J Agron. 2002;17(4):353–68.

131. Piñero, M., Carmen, Pérez‐Jiménez, M., López‐Marín, J. and del Amor Francisco M. Fruit quality of sweet pepper as affected by foliar Ca applications to mitigate the supply of saline water under a climate change scenario. J Sci Food Agric. 2017;98:1071-1078.

132. Wheeler RM, Mackowiak CL, Stutte GW, Yorio NC, Berry WL. Effect of elevated carbon dioxide on nutritional quality of tomato. Adv Sp Res. 1997;20(10):1975–8.

133. Heagle AS, Miller JE, Pursley WA. Growth and Yield Responses of Potato to Mixtures of Carbon Dioxide and Ozone. J Environ Qual [Internet]. 2003 Sep [cited 2025 Jul 24];32(5):1603–10. Available from: https://pubmed.ncbi.nlm.nih.gov/14535300/

134. Behboudian, M.H. and Tod C. Postharvest attributes of `Virosa’ tomato fruit produced in an enriched carbon dioxide environment. HortSci. 1995;30:490-491.

135. Singh S, Bhatia A, Tomer R, Kumar V, Singh B, Singh SD. Synergistic action of tropospheric ozone and carbon dioxide on yield and nutritional quality of Indian mustard (Brassica juncea (L.) Czern.). Environ Monit Assess [Internet]. 2013 Aug [cited 2025 Jul 10];185(8):6517–29. Available from: https://pubmed.ncbi.nlm.nih.gov/23283603/

136. Heagle AS, Miller JE, Pursley WA. Growth and Yield Responses of Potato to Mixtures of Carbon Dioxide and Ozone. J Environ Qual. 2003 Sep;32(5):1603–10.

137. Schonhof I, Kläring HP, Krumbein A, Schreiner M. Interaction between atmospheric CO2 and glucosinolates in broccoli. J Chem Ecol. 2007;33(1):105–14.

138. Jain V, Pal M, Raj A, Khetarpal S. Photosynthesis and nutrient composition of spinach and fenugreek grown under elevated carbon dioxide concentration. Biol Plant. 2007;51(3):559–62.

139. Lieffering M, Kim HY, Kobayashi K, Okada M. The impact of elevated CO2 on the elemental concentrations of field-grown rice grains. F Crop Res [Internet]. 2004 Aug 10 [cited 2025 Jul 9];88(2–3):279–86. Available from: https://www.sciencedirect.com/science/article/abs/pii/S0378429004000164

140. Yang L, Wang Y, Dong G, Gu H, Huang J, Zhu J, et al. The impact of free-air CO2 enrichment (FACE) and nitrogen supply on grain quality of rice. F Crop Res. 2007 Jun 5;102(2):128–40.

141. Köhler IH, Huber SC, Bernacchi CJ, Baxter IR. Increased temperatures may safeguard the nutritional quality of crops under future elevated CO2 concentrations. Plant J [Internet]. 2019 Mar 1 [cited 2025 Jul 7];97(5):872–86. Available from: /doi/pdf/10.1111/tpj.14166

142. Víllora G, Moreno DA, Pulgar G, Romero L. Yield improvement in zucchini under salt stress: Determining micronutrient balance. Sci Hortic (Amsterdam). 2000 Nov 3;86(3):175–83.

143. Wang Y, Song Q, Frei M, Shao Z, Yang L. Effects of elevated ozone, carbon dioxide, and the combination of both on the grain quality of Chinese hybrid rice. Environ Pollut [Internet]. 2014 [cited 2025 Jul 9];189:9–17. Available from: https://pubmed.ncbi.nlm.nih.gov/24607649/

Appendices

Appendix 1: Data tables form data analysis

Appendix 1a: Experimental studies

Appendix 1b: Meta-analysis studies

Appendix 1c: Modelling studies

Appendix 2 : Graphs for individual food groups from experimental studies


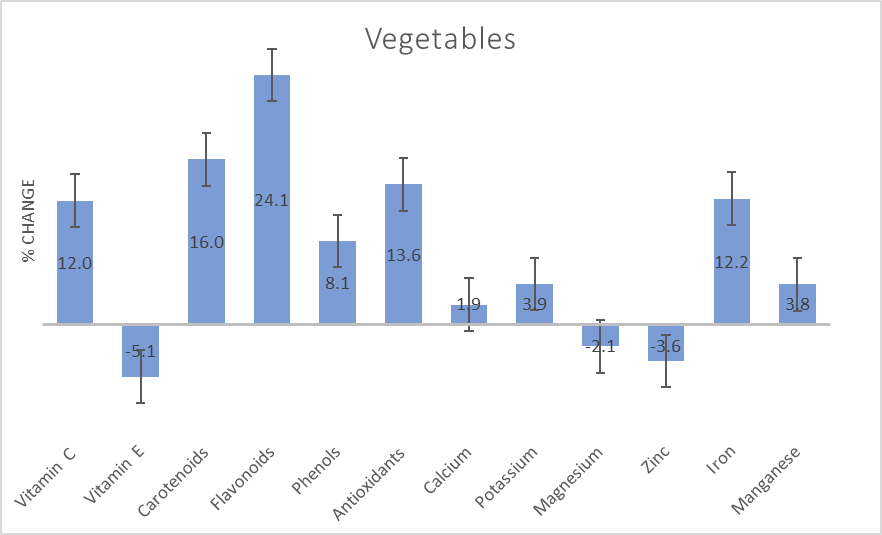


**Figure 1s:** The figure is based on 42 experimental studies of vegetables, legumes, and grains, where both positive and negative climate impacts on nutritional quality parameters were observed. Percentage changes reflect crop responses to elevated exposures: CO₂ (>350 ppm), temperature (+4 °C), ozone (+25%), salinity (+25%), water stress (−50%), and contaminated water. Multiple data points for each nutrient parameter were averaged. Error bars represent the standard error of the mean: phytochemicals (n=59), Ca (n=21), K (n=16), Mg (n=14), Zn (n=23), Fe (n=25), Mn (n=11), P (n=5), S (n=2), and vitamins (n=33), and carbohydrate (n=3). Error margins reflect discordance between field and greenhouse experiments, which were considered jointly.


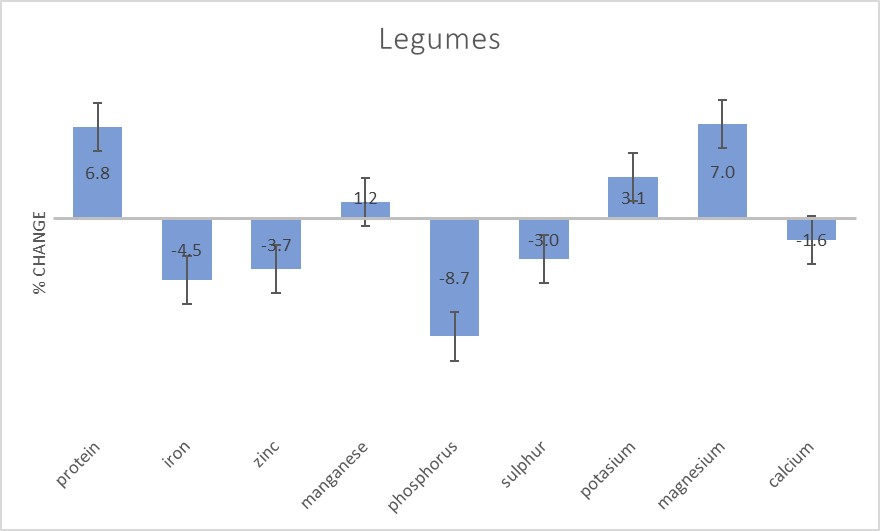


**Figure 2s:** The figure is based on 19 experimental studies of vegetables, legumes, and grains, where both positive and negative climate impacts on nutritional quality parameters were observed. Percentage changes reflect crop responses to elevated exposures: CO₂ (>350 ppm), temperature (+4 °C), ozone (+25%), salinity (+25%), water stress (−50%), and contaminated water. Multiple data points for each nutrient parameter were averaged. Error bars represent the standard error of the mean: Ca (n=3), K (n=1), Mg (n=1), Zn (n=11), Fe (n=11), Mn (n=2), P (n=2), S (n=2), and protein (n=4). Error margins reflect discordance between field and greenhouse experiments, which were considered jointly


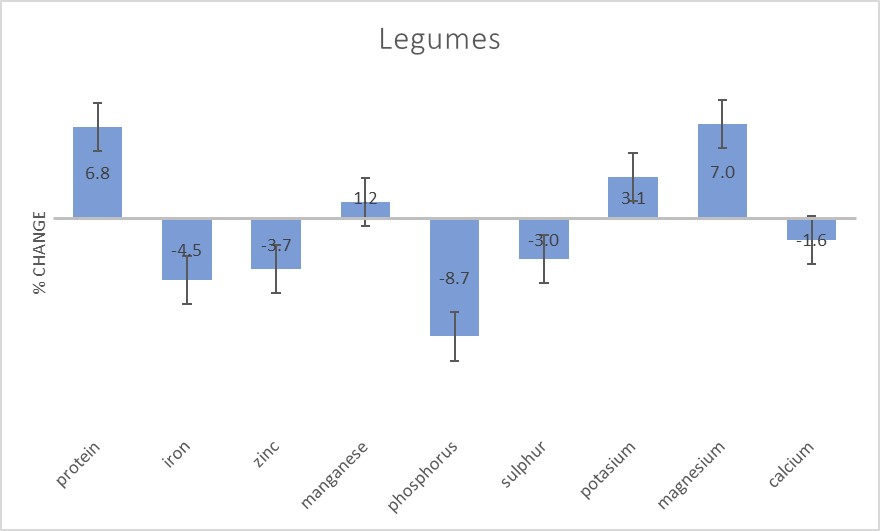


**Figure 3s:** The figure is based on 21 experimental studies of vegetables, legumes, and grains, where both positive and negative climate impacts on nutritional quality parameters were observed. Percentage changes reflect crop responses to elevated exposures: CO₂ (>350 ppm), temperature (+4 °C), ozone (+25%), salinity (+25%), water stress (−50%), and contaminated water. Multiple data points for each nutrient parameter were averaged. Error bars represent the standard error of the mean: Ca (n=5), K (n=5), Mg (n=5), Zn (n=22), Fe (n=20), P (n=3), vitamins (n=1), protein (n=6), and carbohydrate (n=3). Error margins reflect discordance between field and greenhouse experiments, which were considered jointly

Appendix 3: Methodology adopted database search strategies <https://doi.org//10.1073/pnas.1800442115>


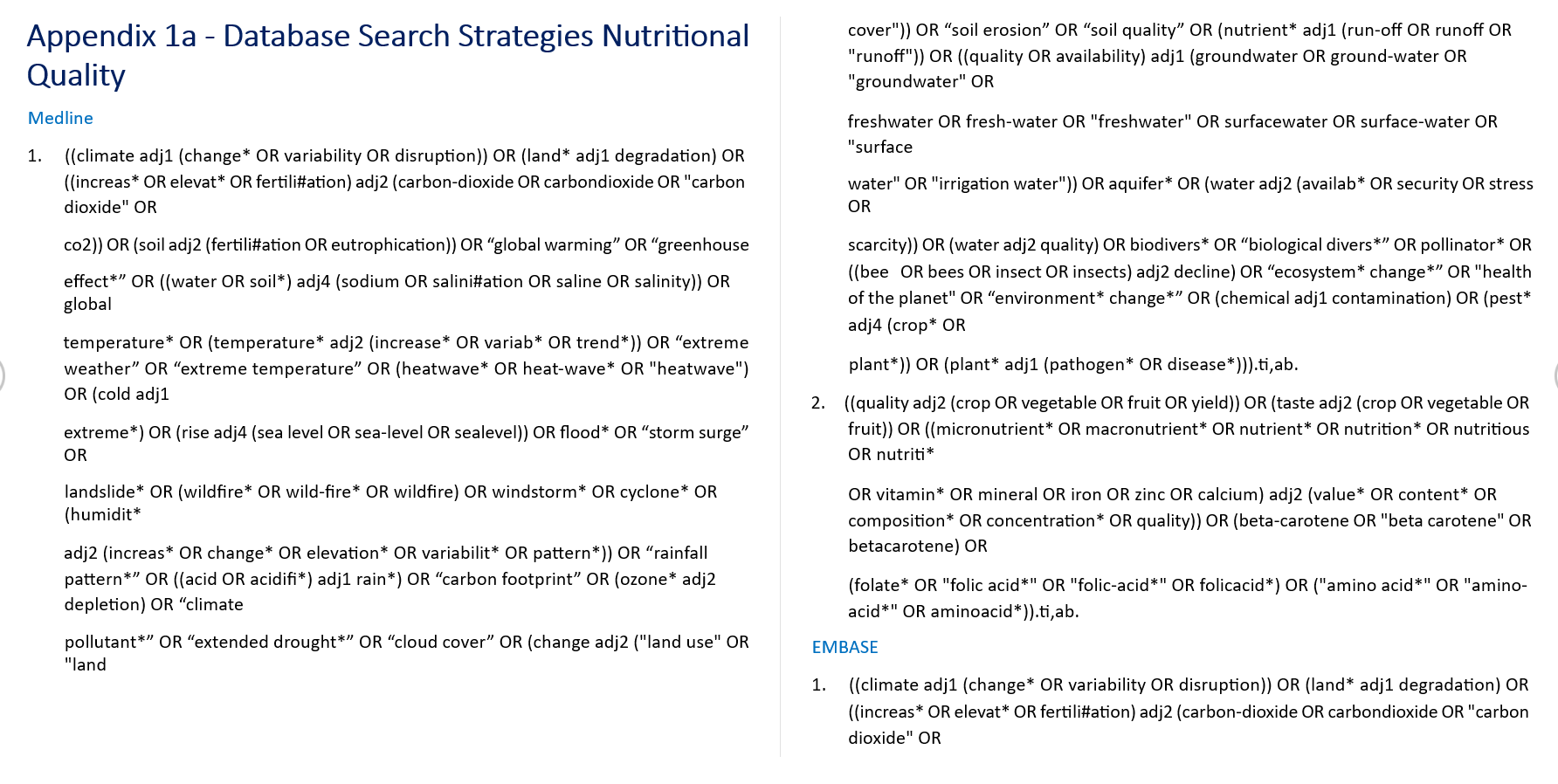


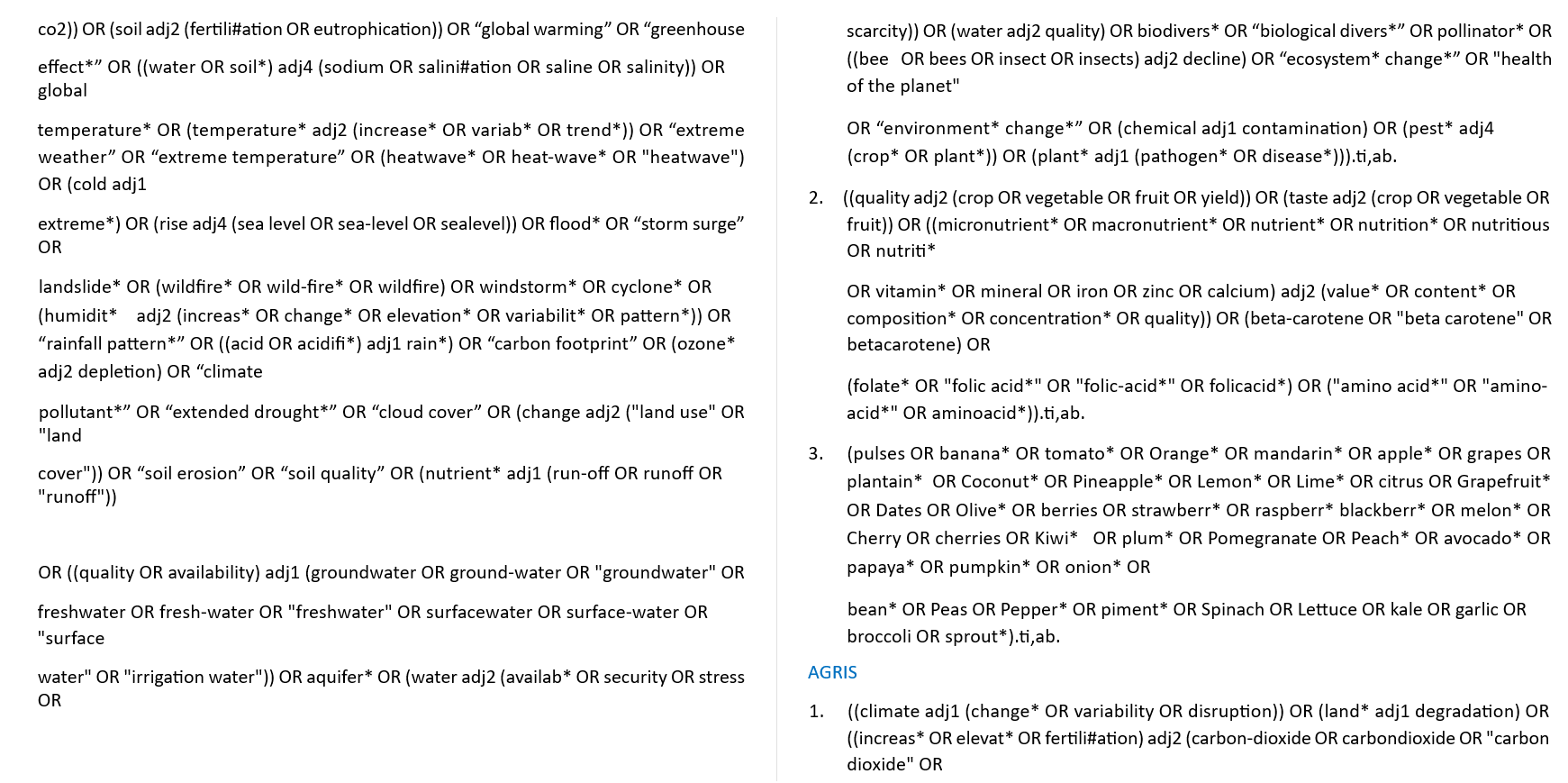


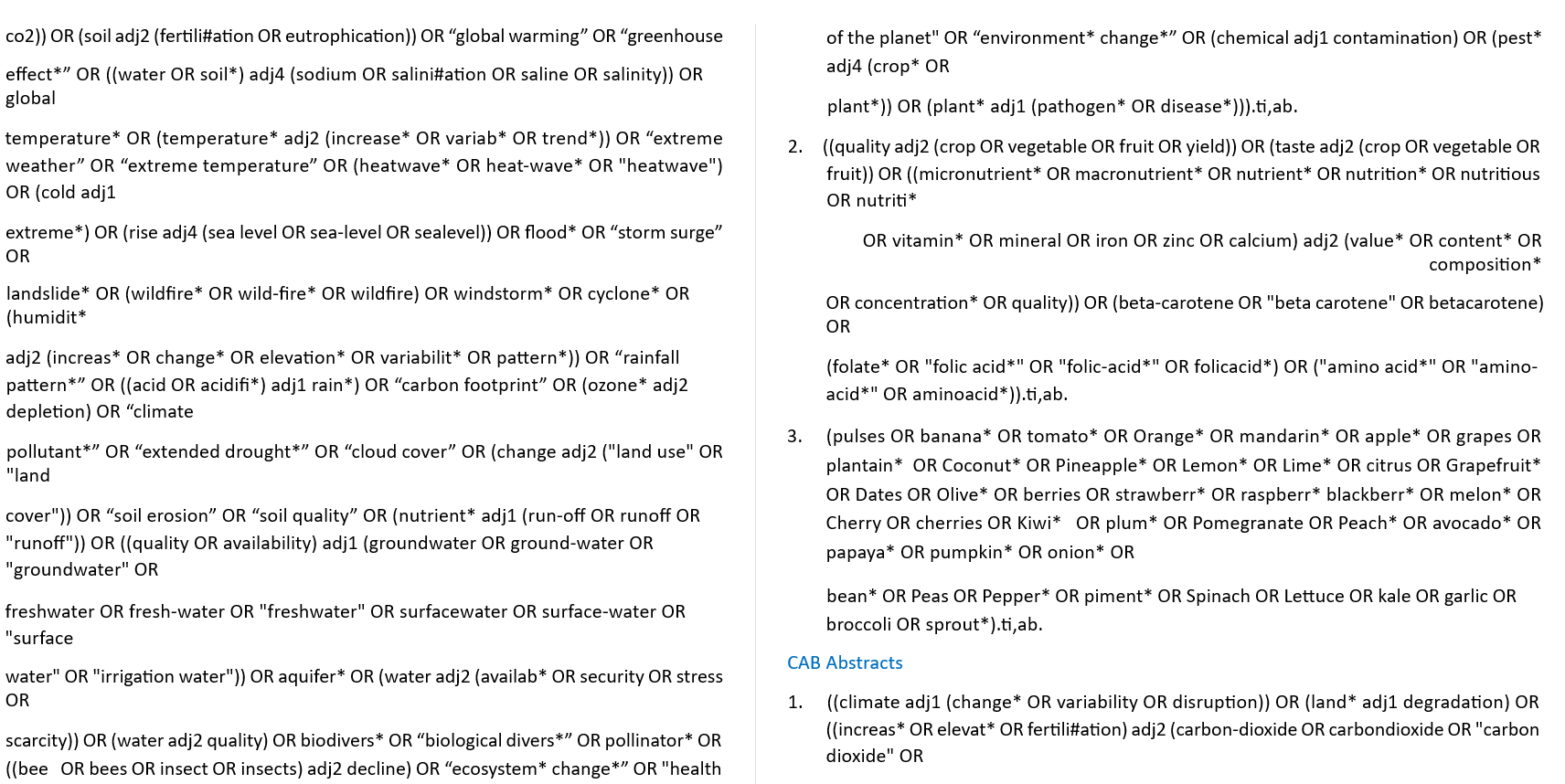


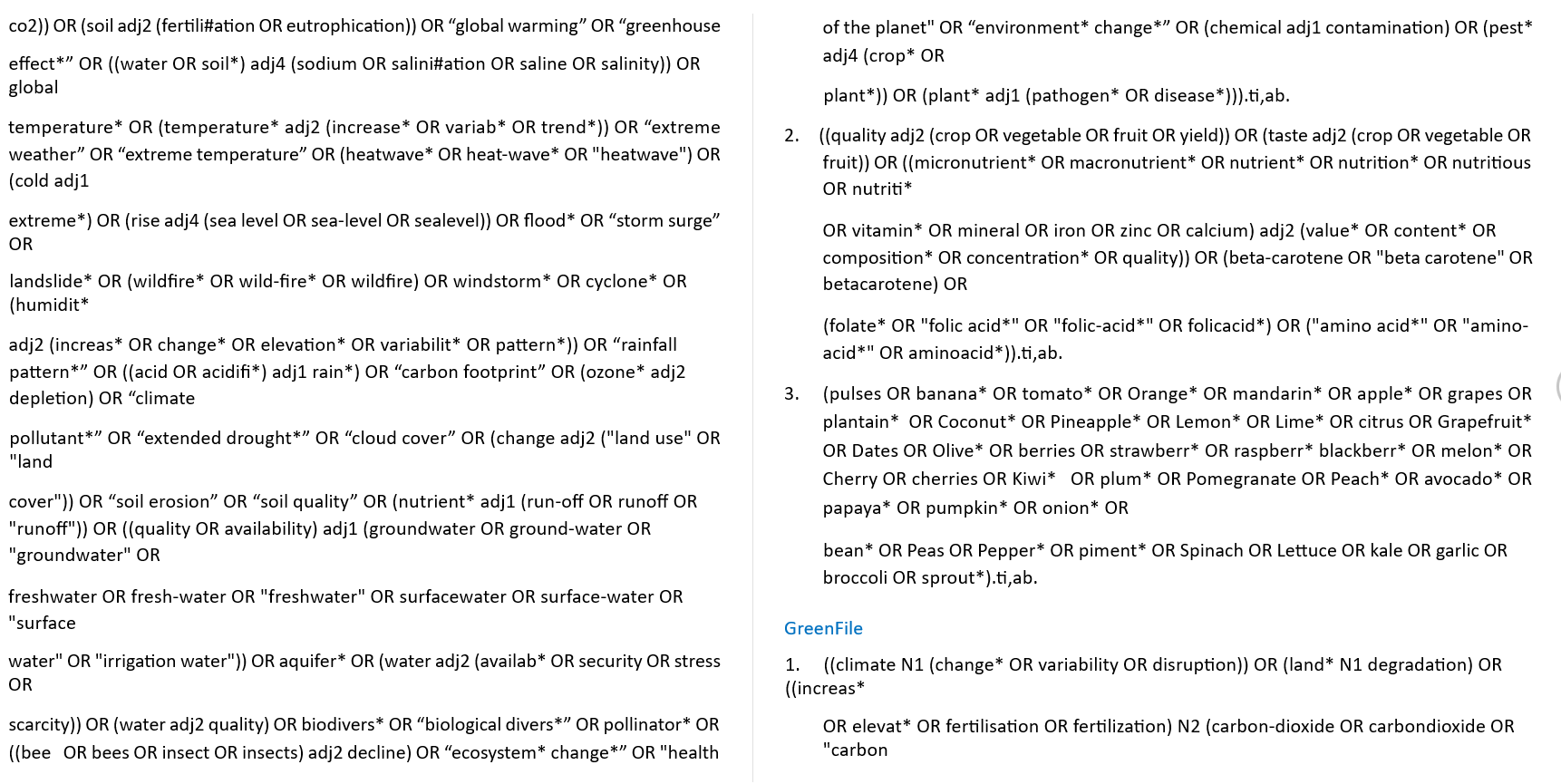


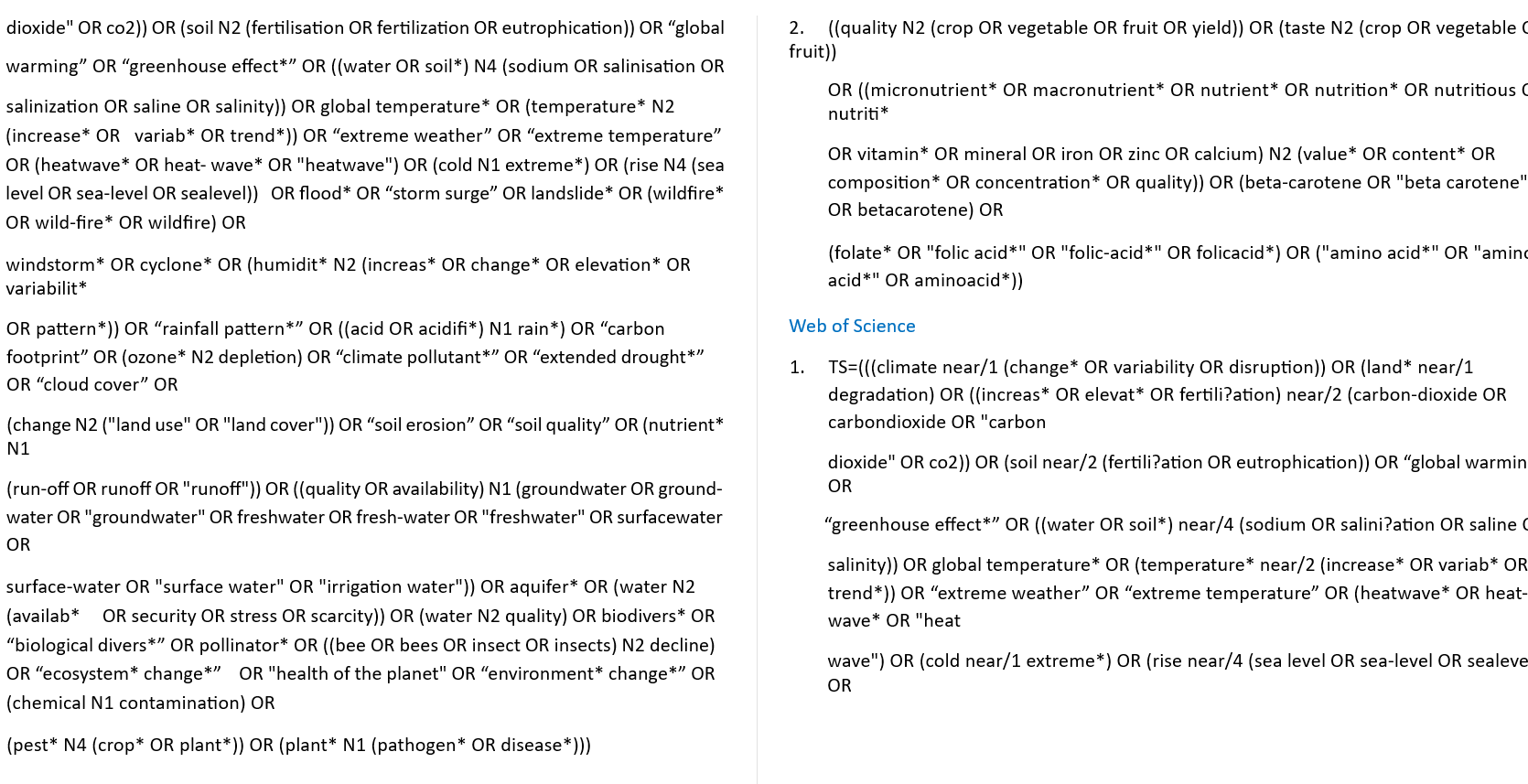


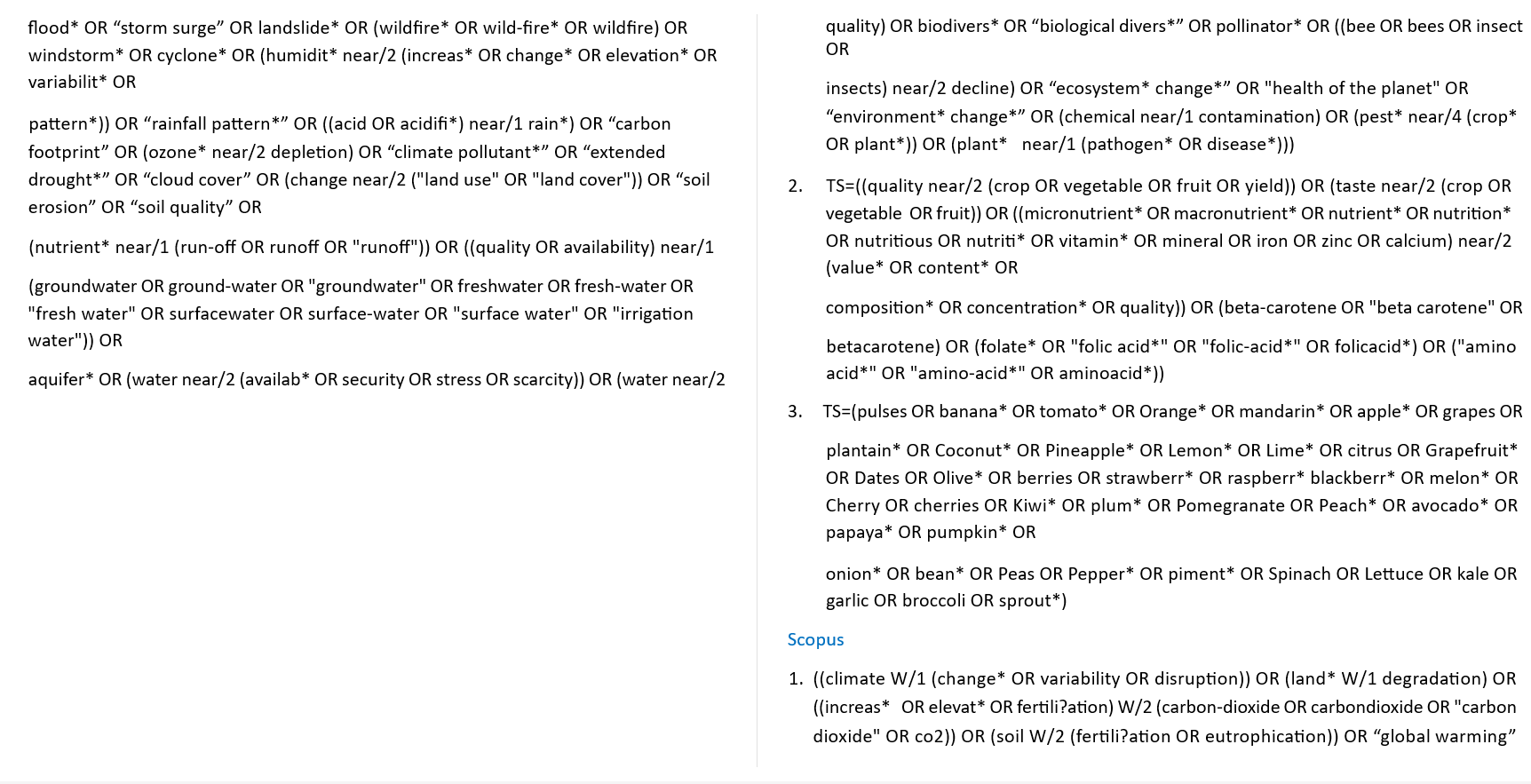


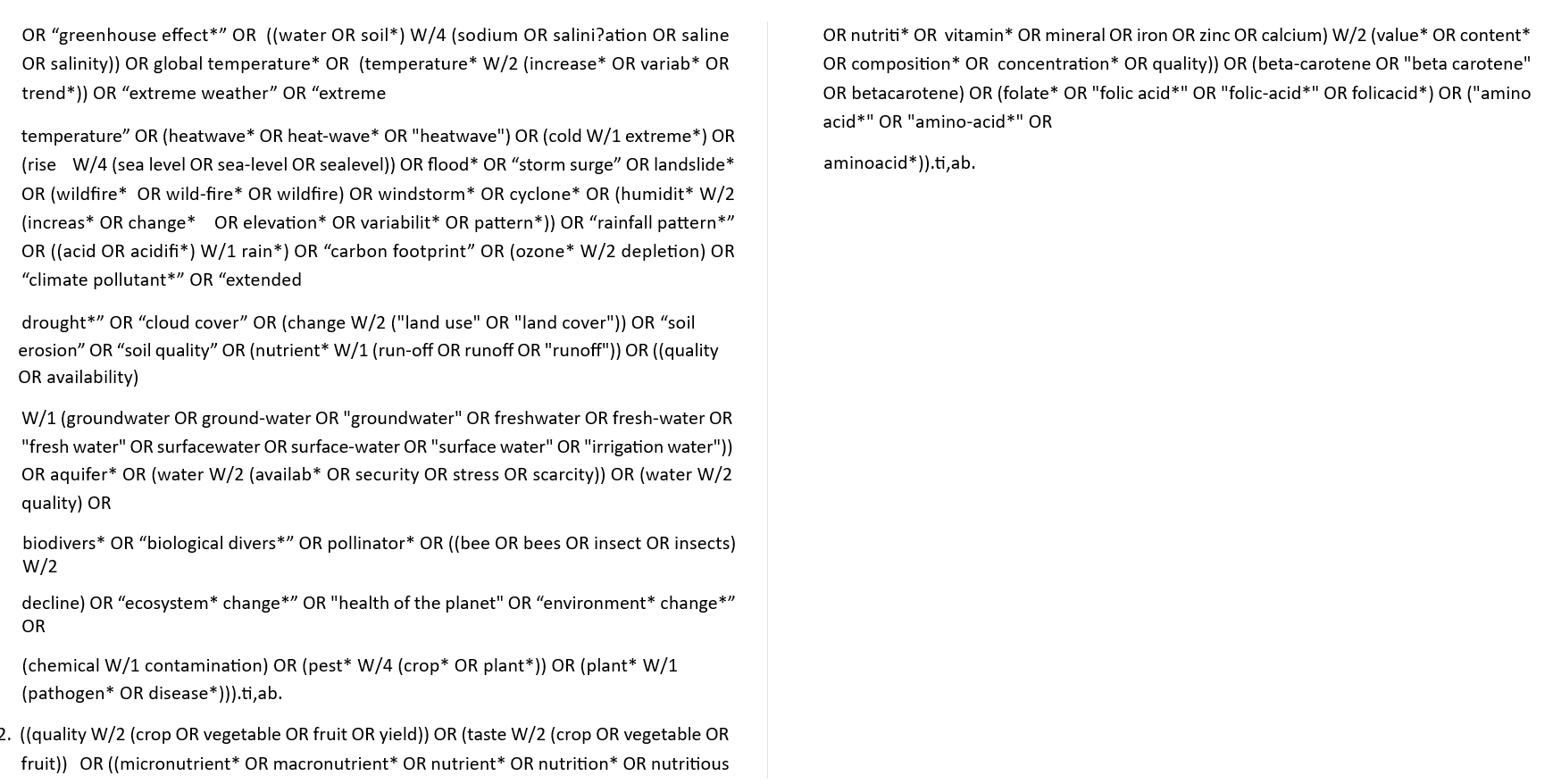


Appendix 4: PRISMA checklist

**
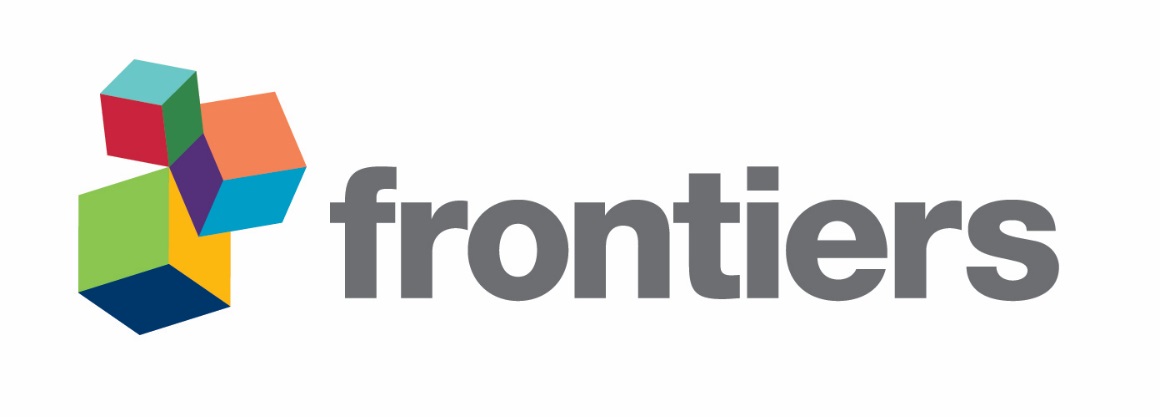
**
